# Hyperplastic and Neoplastic Lesions of the Glandular Stomach and Intestine in Two Inbred Strains of Mice and their Reciprocal Hybrids

**DOI:** 10.1038/bjc.1956.13

**Published:** 1956-03

**Authors:** E. W. Miller, F. C. Pybus

## Abstract

**Images:**


### HYPERPLASTIC AND NEOPLASTIC LESIONS OF THE GLANDULAR STOMACH AND INTESTINE IN TWO INBRED STRAINS OF MICE AND THEIR RECIPROCAL HYBRIDS

E. W. MILLER AND F. C. PYBUS

*From The J. H. Burn Research Laboratory, Royal Victoria Infirmary, Newcastle upon Tyne*

Received for publication December 3, 1955

SPONTANEOUS tumours of the glandular stomach and the intestine are rare in mice and rats. The few published descriptions are mentioned in a valuable review by Klein and Palmer (1941) of the literature on spontaneous and induced neoplasia in these organs. Since that date gastric lesions have been found in Strong's NHO strain of mice and have been described by Strong, Collins and Durand (1943), Strong (1945, 1947), McPeak and Warren (1947), Kaplan (1949) and Smith and Strong (1949); these lesions are of particular interest in connection with the present report, which is concerned with lesions of a precancerous and carcinomatous nature found in the glandular stomach and, rarely, in the intestine of certain mice.

### MATERIAL AND METHODS

As described in detail in the first communication in this series (Miller and Pybus, 1954*a*), mice of the two inbred strains CBA and NBT were crossed reciprocally, and the resulting hybrids were inbred by brother-sister matings for a total of 12 generations, to give the CBA/NBT (or CN) and the NBT/CBA (or NC) strains. When two months old half of the mice in every $F_1$ litter were each given one subcutaneous injection of 1 mg. methylcholanthrene in 0·1 c.c. sesame oil. These injected mice were then inbred for a total of 10 generations, the members of each generation being similarly injected; these formed the M/CN and M/NC groups. After 4 generations, in which every animal was injected, certain litters from $F_5$ onwards (from mice which early developed tumours at the site of injection) were neither injected nor bred from; these, together with 2 uninjected generations $F_{11}$ and $F_{12}$ (raised from the tenth injected generation), formed the M/CN and M/NC *uninjected* groups.

A number of mice from the two inbred parent strains were likewise injected with methylcholanthrene, forming the M/CBA and M/NBT groups, and these injected mice were bred from by brother-sister matings to give one generation of uninjected mice (M/CBA $F_1$ and M/NBT $F_1$).

There were thus 3 groups of reciprocal hybrids, namely the 12 inbred generations of untreated mice, the 10 inbred generations of injected mice, and the 8 generations of untreated mice which were offspring of mice injected for from 4 to 10 generations. There were also 3 groups of the pure strain mice, namely the untreated mice of each pure strain raised during the period of the experiment, the one generation of injected mice, and the one generation of uninjected mice which were offspring of the treated generation. All the untreated groups served as controls for any effect of the carcinogen.

All treated and control mice were fed alike on a diet of rat cubes *ad lib.* (supplied by the North-Eastern Agricultural Co-operative Society, Ltd, of Aberdeen, and manufactured by them to the formula of the Rowett Research Institute) with small daily supplements of fresh carrot and cabbage alternately (and, in season, dandelion leaves), and tap water *ad lib.* This diet was supplemented for the breeding pairs by fresh milk daily, given on brown bread.

Although the experiment began in 1945, no stomachs were opened until December, 1946, when a case of gross epithelioma of the forestomach was observed. Since that date the stomach of every mouse dying in the laboratory has been opened and examined ; all mice in the experiment dying before that date have been omitted from this report. The incidence of forestomach tumours in the present material has already been described (Miller and Pybus, 1955). The stomach was never distended before fixation ; it was opened by a longitudinal incision along the greater curvature, laid out flat, gently cleaned of any contents and carefully examined. All nodules and any suspicious-looking regions were then excised together with surrounding tissues and fixed in Susa ; sections were cut at $6\mu$ and stained with Ehrlich's haematoxylin and eosin.

*Morphology and histology of stomach lesions*

The mouse stomach consists of two parts. the forestomach lined by squamous epithelium and the pyloric stomach containing the digestive glands, the two regions being separated by a limiting ridge of squamous epithelium.

As a result of the routine examination of every stomach many cases of pathological lesions in the glandular portion were seen which would otherwise have escaped notice, as very often there was no external indication of their presence. Sometimes a small dimple or a deeper indentation on the outer surface was evident, and in a few cases gross distension of the duodenum occurred to a size at least as great as the normal stomach. In only one instance (uninjected mouse M/NC $F_{10}$/297) was there a definite lump on the external wall.

In the opened stomach the lesions were visible as white or translucent nodules of various sizes from the tiniest nodule to a tumour the size of a pea or larger. More rarely there would be a diffuse thickened area, not of great extent. The nodules were most usually solitary, but sometimes 2 or (rarely) 3 were found, and in one mouse there were 4. They occurred at any part of the glandular stomach, in the fundic portion near the limiting ridge, on greater or lesser curvature, and often close to the pylorus. In this last position there were often 2 symmetrically-placed nodules, usually of the same size, or a third smaller nodule might be present ; the maximum number seen in this region was also 4. Table I shows the frequency of multiple nodules and also the distribution between the pyloric and fundic regions. A noticeable feature was the high proportion in the CN hybrids of 2 symmetrical nodules at the pyloric opening.

At no time was there seen a thickened zone forming a complete ring round the pyloric opening such as was described by Strong (1945) and by Stewart, Hare and Bennett (1953), nor was there any morphological resemblance to the Strain I lesion (Stewart and Andervont, 1938 ; Andervont, 1939, 1949 ; Stewart, 1940, 1941).

TABLE I.—*Frequency of Multiple Nodules in Glandular Stomachs of Mice of Two Reciprocal-hybrid Strains.*

| | Number of mice showing given number of nodules per individual. | | | | | | | | | | |
|---|---|---|---|---|---|---|---|---|---|---|---|
| | Pyloric region. | | | | | Fundic region | | | | | |
| | | Number of nodules. | | | | | Number of nodules. | | | | |
| Strain. | Diffuse thickening. | 1 | 2 | 3 | 4 | Diffuse thickening. | 1 | 2 | 3 | 4 | Total. |
| CN | 2 | 7 | 11 | 1 | 0 | 3 | 8 | 1 | 1 | 0 | 34 |
| NC | 0 | 5 | 3 | 0 | 1 | 2 | 10 | 3 | 1 | 0 | 25 |
| M/CN | 0 | 0 | 0 | 0 | 0 | 7 | 1 | 0 | 0 | 0 | 8 |
| M/NC | 0 | 0 | 0 | 0 | 0 | 7 | 3 | 1 | 0 | 0 | 11 |
| M/CN uninjected | 0 | 2 | 0 | 0 | 0 | 4 | 0 | 0 | 0 | 0 | 6 |
| M/NC uninjected | 0 | 14 | 3 | 0 | 0 | 12 | 63 | 9 | 2 | 2 | 105 |
| Total | 2 | 28 | 17 | 1 | 1 | 35 | 85 | 14 | 4 | 2 | 189 |

The polypoid tumours could be sessile or pedunculate ; if the latter, and originating close to the pylorus, they would hang down into the duodenum, nearly blocking the lumen and causing the duodenal distension described above.

The following appeared to be the stages of development as seen in microscopic preparations of the lesions.

(1) Areas of diffuse thickening were usually caused by hyperplasia of the glandular mucosa. The tissues retained their orderly arrangement, but the mucosa was greatly thickened and often folded into deeper corrugations than normal. Foci of atypical cells, and, occasionally, multiple cysts were to be seen in the mucosa.

(2) In the adenomatous nodule there was a disorderly arrangement of the mucosal cells to form acini and cysts ; the tumour was sometimes sessile, but at other times had a definite stalk (Fig. 1). The neighbouring epithelium was quite normal but, beneath the tumour, the submucosa was usually infiltrated by lymphocytes and elevated into a projection into the base of the tumour, thus causing the visible indentation in the outer surface of the stomach wall.

(3) At the bases of many adenomata there was a downgrowth of the mucosal cells through the muscularis mucosae. This varied from the passage of a single acinus or 2 or 3 acini (Fig. 2) to penetration along quite an extensive front, completely disrupting the muscularis mucosae. In the submucosa the invading epithelium spread out to form acini and cysts which could be lined by an orderly arrangement of epithelial cells, but in which there were often projecting papillae (Fig. 2). Strong, Collins and Durand (1943) classified this stage as adenocarcinoma or potential adenocarcinoma to distinguish it from the benign adenoma or papilloma where such invasion was absent.

In one mouse in which there were 2 symmetrical pyloric nodules, one was an adenoma and the other a potential adenocarcinoma (by the above definition), and the neighbouring Brunner's glands had become adenomatous (Fig. 3). Christie (1953) described the carcinomatous transformation of Brunner's glands in a case of human duodenal carcinoma.

(4) While the stage of submucosal extension was comparativeiy common, the next stage, penetration of the circular muscle, was much rarer. In the few cases where this was observed it appeared that one acinus or a few acini were passing out through the muscle in the line of entry of a blood vessel, but it was not considered that the epithelial cells were within the blood vessel (Fig. 4). No extensive penetration on a broad front through the circular muscle was seen, and the few acini arriving at the surface were still covered by the serosa and in one or two instances appeared to be becoming fibrosed (Fig. 5).

TABLE II.—*The Incidence of the Successive Stages of Neoplasia in Glandular Gastric Lesions in Mice of Two Reciprocal-hybrid Strains.*

| | Number of mice showing each stage. | | | | | |
|---|---|---|---|---|---|---|
| | | | Adenocarcinoma. | | | |
| Strain. | Hyperplasia. | Adenoma. | Penetration of muscularis mucosae. | Penetration of circular muscle. | Carcinoma. | Total. |
| CN | 3 | 9 | 22 | 0 | 0 | 34 |
| NC | 1 | 15 | 7 | 2 | 0 | 25 |
| M/CN | 7 | 0 | 1 | 0 | 0 | 8 |
| M/NC | 6 | 1 | 4 | 0 | 0 | 11 |
| M/CN uninjected | 2 | 2 | 2 | 0 | 0 | 6 |
| M/NC uninjected | 11 | 57 | 28 | 8 | 1 | 105 |
| Total | 30 | 84 | 64 | 10 | 1 | 189 |

TABLE III.—*The average Ages at Death of Mice Showing Each Successive Stage of Neoplasia in Glandular Gastric Lesions in Two Reciprocal-hybrid Strains.*

| | Average ages for each stage (in months). | | | | |
|---|---|---|---|---|---|
| | | | Adenocarcinoma. | | |
| Strain. | Hyperplasia. | Adenoma. | Penetration of muscularis mucosae. | Penetration of circular muscle. | Carcinoma. |
| CN | 17·8 | 24·2 | 20·2 | — | — |
| NC | 20·0 | 24·9 | 21·4 | 15·3 | — |
| M/CN | 11·9 | — | 19·5 | — | — |
| M/NC | 12·0 | 18·0 | 19·4 | — | — |
| M/CN uninjected | 13·8 | 15·0 | 20·0 | — | — |
| M/NC uninjected | 19·9 | 22·1 | 23·6 | 23·0 | 26·0 |

The numbers of mice showing each of these stages of neoplasia are shown in Table II. Table III gives the average age, for each group of hybrids, of the mice showing each stage ; on the whole, hyperplasia was seen in the younger and adenocarcinoma in the older mice, but there was a good deal of overlapping.

To all these stages the term precancer was given by Stewart and Lorenz (1942) and by Stewart, Hare, Lorenz and Bennett (1949). Stewart and Lorenz (1949) introduced the term " nearocarcinoma " (of Greek derivation) in preference to " precancerous " to denote " early changes associated with developing carcinoma "; true carcinoma was defined as a neoplasm extending through all the muscle layers on to the serosa. The criteria of induced malignancy were defined by Klein and Palmer (1941) ; by these criteria none of the present lesions can be considered malignant as no metastases were seen and there were no instances of invasion of neighbouring tissues. But by the definition of Stewart and Lorenz (1949) one of the lesions may have been malignant (mouse M/NC $F_{10}$/297, uninjected) ; this tumour (Fig. 6), an oxyntic cell carcinoma, arose near the limiting ridge between the glandular and non-glandular regions and consisted of cystic glandular tissue lying in a groundwork of acidophilic (oxyntic) cells, some of which had very large nuclei (Fig. 7). The tumour cells passed through all the muscle layers, but still appeared to be covered by the unbroken serous membrane ; the tumour cells also penetrated the squamous epithelium. There were no metastases and there was no infiltration of surrounding tissues.

*Morphology and histology of intestinal lesions*

In addition to the gastric lesions just described, there were 7 cases of duodenal neoplasia in 5 females and 2 males, aged 16 to 26 months ; one adenoma of the rectum and one potential adenocarcinoma of the colon in a female aged 20 months, and one intestinal polyp in a 13-months-old female.

The duodenal tumours, sessile or pedunculate, varied in size from a pin-head to a small bean, the latter causing distension to the size of a stomach. Histologically they resembled the gastric lesions, being either simple adenomata with no penetration of the muscularis mucosae, or potential adenocarcinomata with a disorderly arrangement of atypical glands and slight or extensive penetration of the muscularis mucosae ; Brunner's glands were unaltered (Fig. 8). In the most extensive lesion the intestinal wall was altered for a distance of 10 mm.

The colon tumour was the size of a small pea and contained large cysts lined with epithelium and containing mucus-secreting cells. There was extensive epithelial penetration of the muscularis mucosae and widespread submucosal infiltration by leucocytes, with surface ulceration and granulomatous development of connective tissue. The rectal adenoma was the size of a large pea and protruded at the anus ; there was slight epithelial penetration of the muscularis mucosae, and, like the colon tumour, there was surface ulceration and granulomatous development of connective tissue (Fig. 9).

The intestinal polyp was a pedunculate adenoma, with disorderly arrangement of glandular epithelium ; goblet cells were present and the epithelium had penetrated into the submucosa of the stalk.

*The incidence of glandular gastric lesions in the inbred parent strains*

Spontaneous pathological lesions of the glandular stomach were very rare in the two inbred parent strains. One case of spontaneous glandular carcinoma of the stomach was seen in 1938 in an 18-months-old $F_9$ female of the NBT strain, of the line from which all the present experimental mice were descended.

During the period of the experiment, in 72 NBT mice aged 17 months and over, of which the stomachs were examined after December, 1946, there were 3 cases of stomach lesions, the earliest in a mouse aged 17 months. One $F_{34}$ mouse had a simple adenoma, one in $F_{35}$ had 2 tumours near the pylorus with epithelial penetration of the muscularis mucosae, and the third ($F_{36}$) had an adenoma plus diffuse changes in the mucosa and slight penetration of the muscularis mucosae. There was a certain degree of relationship between the 3 mice ; all belonged to Sub-line C and were descended from the same $F_{28}$ pair, and the sister of the $F_{34}$ female was the grandmother of the $F_{36}$ mouse. It is impossible to say what the incidence of the lesion was in the strain prior to December, 1946, but the gross case in 1938 shows that the character was present in $F_9$.

In the CBA strain only one case was seen in 413 mice aged 17 months and over during the period of the experiment, in an $F_{35}$ male which had an adenomatous nodule with epithelial penetration of the muscularis mucosae at the base of the stalk. Since the experiment ended one more case has been seen, in an $F_{41}$ male, in a sub-line separate from that of the first case since $F_1$. Neither case was related to Sub-lines P or Q, but the second instance was descended in Sub-line R ; these were the three sub-lines used for hybridising in the present experiment (Miller and Pybus, 1954*a*).

In the M/NBT group, injected with methylcholanthrene, there were 2 cases in an effective total of 26 mice, namely an adenoma in an $F_{29}$ male and an adenoma in his $F_{30}$ niece. (In the analysis of the incidence of these lesions only the effective total of mice in each group is considered. i.e. the number which survived to the age at which the earliest lesion was found.)

In the uninjected offspring of the M/NBT mice (i.e. M/NBT $F_1$), there was one case of glandular hyperplasia near the pylorus in a 13-months-old male. This mouse and the 2 M/NBT mice were likewise descended from the $F_{28}$ pair mentioned above. Litter-mates of this same pair were the Sub-line C mice used for hybridising in the present experiment.

No lesions were found in the injected M/CBA group, but there was one case amongst their uninjected offspring (M/CBA $F_1$)—an adenoma with early epithelial penetration of the muscularis mucosae in a female belonging to Sub-line Q.

Table IV shows the incidences of glandular stomach lesions in these six groups of pure strain mice. There was no statistically significant difference between the incidences in the two sexes in any group. Comparison of the incidences in the two strains, in the control and treated mice of each strain, in the treated mice and their untreated ($F_1$) offspring, and in the controls and the untreated $F_1$ mice also showed no significant difference.

TABLE IV.—*Data for Lesions in Glandular Stomach in Control Mice of Pure Strains NBT and CBA, in Mice of these Strains Treated with Methylcholanthrene (M/NBT and M/CBA), and in the Uninjected Generation Descended from the Treated Mice (M/NBT $F_1$ and M/CBA $F_1$).*

| | | | | Affected mice. | | | | | | Unaffected mice. | | |
|---|---|---|---|---|---|---|---|---|---|---|---|---|
| | | | | | | | Age at death (months). | | | Age at death (months). | | |
| Strain. | Sex. | Earliest lesion (months). | Effective number of mice. | No. | Percentage. | | Range. | Average. | | Range. | Average. | |
| NBT | F | 17·0 | 59 | 2 | 3·4 | 4·2 | 17·0–18·5 | 17·8 | 17·5 | 17·0–24·0 | 18·8 | 18·6 |
| | M | 17·0 | 13 | 1 | 7·7 | | 17·0 | 17·0 | | 17·0–22·0 | 17·6 | |
| CBA | F | — | 211 | 0 | 0·0 | 0·2 | — | — | 17·0 | 17·0–44·0 | 25·3 | 23·9 |
| | M | 17·0 | 202 | 1 | 0·5 | | 17·0 | 17·0 | | 17·0–34·0 | 22·4 | |
| M/NBT | F | 18·5 | 21 | 1 | 4·8 | 7·7 | 18·5 | 18·5 | 15·0 | 11·0–17·5 | 14·7 | 14·2 |
| | M | 11·5 | 5 | 1 | 20·0 | | 11·5 | 11·5 | | 11·0–12·5 | 11·6 | |
| M/CBA | F | — | 12 | 0 | 0·0 | 0·0 | — | — | — | 12·5–27·0 | 17·0 | 17·5 |
| | M | — | 14 | 0 | 0·0 | | — | — | | 12·5–26·0 | 18·0 | |
| M/NBT $F_1$ | F | — | 64 | 0 | 0·0 | 0·9 | — | — | 13·0 | 13·0–22·5 | 17·1 | 16· |
| | M | 13·0 | 45 | 1 | 2·2 | | 13·0 | 13·0 | | 13·0–19·0 | 15·1 | |
| M/CBA $F_1$ | F | 22·5 | 151 | 1 | 0·7 | 0·5 | 22·5 | 22·5 | 22·5 | 22·0–34·0 | 26·4 | 25·6 |
| | M | — | 68 | 0 | 0·0 | | — | — | | 22·0–28·0 | 24·4 | |

TABLE V.—*Data for Lesions in Glandular Stomach in Mice of the Three Groups, NBT, M/NBT and M/NBT $F_1$, belonging to Sub-line C.*

| Strain. | Sex. | Earliest lesion (months). | Effective number of mice. | Affected mice. No. | Percentage. | | Age at death (months). Range. | Average. | | Unaffected mice. Age at death (months). Range. | Average. | |
|---|---|---|---|---|---|---|---|---|---|---|---|---|
| NBT | F | 17·0 | 36 | 2 | 5·6 | 6·8 | 17·0–18·5 | 17·8 | 17·5 | 17·0–21·0 | 18·7 | 18·5 |
| | M | 17·0 | 8 | 1 | 12·5 | | 17·0 | 17·0 | | 17·0–22·0 | 17·8 | |
| M/NBT | F | 18·0 | 12 | 1 | 8·3 | 13·3 | 18·5 | 18·5 | 15·0 | 11·0–17·5 | 14·4 | 13·8 |
| | M | 11·5 | 3 | 1 | 33·3 | | 11·5 | 11·5 | | 11·0–11·0 | 11·0 | |
| M/NBT $F_1$ | F | — | 34 | 0 | 0·0 | 1·7 | — | — | 13·0 | 13·0–22·5 | 16·8 | 16·2 |
| | M | 13·0 | 26 | 1 | 3·8 | | 13·0 | 13·0 | | 13·0–19·0 | 15·3 | |

TABLE VI.—*Data for Spontaneous Lesions in Glandular Stomach and Duodenum in CN Group of Hybrids. Earliest Lesions at* 14 *Months.*

| Generation and sex | | Effective number of mice. | Affected mice. No. | Age at death (months). Range. | Average. | | Unaffected mice. Age at death (months). Range. | Average. | | Type of cross. |
|---|---|---|---|---|---|---|---|---|---|---|
| $F_1$ | F | 54 | 3 | 26·5–29·0 | 27·7 | 27·3 | 17·0–34·0 | 26·9 | 25·2 | 3QB |
| | M | 52 | 1 | 26·0 | 26·0 | | 17·0–31·0 | 23·5 | | 1PC |
| $F_2$ | F | 131 | 1 | 22·5 | 22·5 | 23·3 | 14·0–32·0 | 24·3 | 23·1 | PC |
| | M | 105 | 3 | 21·0–25·0 | 23·5 | | 14·0–31·0 | 21·5 | | |
| $F_3$ | F | 33 | 3 | 24·0–26·0 | 25·3 | 23·2 | 16·0–32·0 | 24·7 | 22·8 | 2PC<br>1QA<br>2QB |
| | M | 26 | 2 | 17·0–23·0 | 20·0 | | 14·0–28·0 | 20·5 | | |
| $F_4$ | F | 28 | 1 | 28·0 | 28·0 | 22·5 | 17·0–29·0 | 23·4 | 21·6 | 1PC<br>1QB<br>1QA |
| | M | 23 | 2 | 17·5–22·0 | 19·8 | | 14·0–28·0 | 19·3 | | |
| $F_5$ | F | 23 | 4* | 24·0–27·0 | 25·7 | 24·9 | 15·0–33·0 | 24·7 | 22·2 | 3PC<br>1QA<br>2QB |
| | M | 15 | 2 | 17·0–29·0 | 23·0 | | 14·0–29·0 | 18·5 | | |
| $F_6$ | F | 23 | 1 | 21·0 | 21·0 | 21·0 | 14·0–27·0 | 21·0 | 20·7 | QB |
| | M | 1 | 0 | — | — | | 14·0 | 14·0 | | |
| $F_7$ | F | 18 | 0 | — | — | — | 14·0–30·0 | 20·0 | 18·4 | — |
| | M | 12 | 0 | — | — | | 14·0–24·0 | 16·1 | | |
| $F_8$ | F | 33 | 1 | 20·0 | 20·0 | 20·0 | 14·0–28·0 | 21·7 | 20·7 | PC |
| | M | 10 | 0 | — | — | | 14·0–23·0 | 17·5 | | |
| $F_9$ | F | 34 | 0 | — | — | — | 14·0–32·0 | 23·1 | 22·8 | — |
| | M | 1 | 0 | — | — | | 14·0 | 14·0 | | |
| $F_{10}$ | F | 39 | 0 | — | — | — | 14·0–28·0 | 20·6 | 20·3 | — |
| | M | 2 | 0 | — | — | | 14·0–18·0 | 16·0 | | |
| $F_{11}$ | F | 36 | 1 | 14·0 | 14·0 | 14·0 | 14·0–24·0 | 18·3 | 18·1 | QA |
| | M | 2 | 0 | — | — | | 14·0–15·0 | 14·5 | | |
| $F_{12}$ | F | 83 | 4 | 20·0–24·0 | 21·4 | 17·5 | 14·0–29·0 | 19·0 | 17·6 | QA |
| | M | 69 | 8 | 14·5–17·0 | 15·6 | | 14·0–19·0 | 15·7 | | |
| Total | F | 535 | 19 | 14·0–29·0 | 23·9 | 21·6 | 14·0–34·0 | 22·5 | 21·5 | — |
| | M | 318 | 18 | 14·5–29·0 | 19·3 | | 14·0–31·0 | 19·5 | | |

* Includes 3 mice with duodenal lesions.

The average age at death of the unaffected mice was greater than that of the affected mice except in M/NBT, where the tumour female was the longest-lived.

Since all the NBT, M/NBT and M/NBT $F_1$ cases occurred in Sub-line C, the incidence in mice of that line only in each group was analysed, as shown in Table V. While the incidence in each group was raised (although not significantly so), the differences between the three groups (owing to the small numbers involved) were still not significant, the difference being always less than twice the standard error.

TABLE VII.—*Data for Spontaneous Lesions in Glandular Stomach in NC Group of Hybrids. Earliest Lesion at* 12 *Months.*

| Generation and sex. | Effective number of mice. | Affected mice. No. | Affected mice. Age at death (months). Range. | Affected mice. Age at death (months). Average. | | Unaffected mice. Age at death (months). Range. | Unaffected mice. Age at death (months). Average. | | Type of cross. |
|---|---|---|---|---|---|---|---|---|---|
| $F_1$ F | 57 | 1 | 28·0 | 28·0 | 29·8 | 15·0–36·0 | 26·7 | 24·7 | AQ |
| M | 38 | 1 | 31·5 | 31·5 | | 13·0–31·0 | 21·6 | | DR |
| $F_2$ F | 105 | 3 | 27·0–29·0 | 28·0 | 26·0 | 13·0–31·0 | 23·7 | 22·6 | BR, DR, CP 3BR |
| M | 114 | 3 | 23·5–25·0 | 24·0 | | 12·0–32·0 | 21·7 | | |
| $F_3$ F | 50 | 4 | 23·0–26·0 | 24·8 | 24·8 | 13·0–30·0 | 24·4 | 22·6 | 3BR |
| M | 28 | 0 | — | — | | 12·0–31·0 | 19·6 | | 1DR |
| $F_4$ F | 56 | 6 | 20·0–25·0 | 22·7 | 22·7 | 15·0–29·0 | 23·6 | 20·9 | 5BR |
| M | 42 | 0 | — | — | | 12·0–25·0 | 17·7 | | 1AQ |
| $F_5$ F | 38 | 1 | 25·0 | 25·0 | 25·0 | 14·0–27·0 | 22·1 | 20·0 | CP |
| M | 42 | 0 | — | — | | 12·0–24·0 | 18·1 | | |
| $F_6$ F | 43 | 0 | — | — | — | 12·0–25·0 | 19·7 | 18·4 | — |
| M | 22 | 0 | — | — | | 12·0–21·0 | 15·9 | | |
| $F_7$ F | 22 | 1 | 22·0 | 22·0 | 20·2 | 16·0–28·0 | 20·1 | 18·2 | CP |
| M | 36 | 2 | 18·5–20·0 | 19·8 | | 12·0–23·0 | 17·1 | | CP, DR |
| $F_8$ F | 45 | 0 | — | — | 13·3 | 14·0–27·0 | 20·3 | 18·3 | 3DR |
| M | 32 | 3 | 12·0–14·5 | 13·3 | | 12·0–23·0 | 15·1 | | |
| $F_9$ F | 32 | 0 | — | — | — | 12·0–22·0 | 18·2 | 16·9 | — |
| M | 24 | 0 | — | — | — | 12·0–20·0 | 15·2 | | |
| $F_{10}$ F | 26 | 0 | — | — | — | 12·0–29·0 | 19·0 | 17·8 | — |
| M | 12 | 0 | — | — | | 12·0–19·0 | 15·1 | | |
| $F_{11}$ F | 19 | 0 | — | — | — | 12·0–24·0 | 18·1 | 18·0 | — |
| M | 1 | 0 | — | — | | 15·0 | 15·0 | | |
| $F_{12}$ F | 27 | 0 | — | — | — | 12·0–23·0 | 15·3 | 15·5 | — |
| M | 15 | 0 | — | — | | 12·0–19·0 | 15·9 | | |
| Total F | 520 | 16 | 20·0–29·0 | 24·6 | 23·0 | 12·0–36·0 | 21·9 | 20·5 | — |
| M | 406 | 9 | 12·0–31·5 | 20·2 | | 12·0–32·0 | 18·7 | | |

*The incidence of glandular gastric and intestinal lesions in the untreated reciprocal hybrids, CN and NC*

For the purposes of the analysis of the hybrid groups the intestinal lesions described earlier in this paper are included with the gastric lesions.

In the 12 generations of CN hybrids there were 16 females with lesions in the glandular stomach and 3 with duodenal lesions in a total of 535 females (3·6 per cent) and 18 males with glandular stomach lesions in a total of 318 males (5·7 per cent). The incidences in the two sexes were statistically the same, whether the duodenal tumours were included or not, and the total incidence was 4·3 per cent (Table VI). The average age at death of unaffected mice was slightly less than that of affected mice in the first 6 generations, but slightly greater in the last generations. The last column of Table VI shows the distribution of the lesions in the descendants of the various types of cross between Sub-lines P, Q and R of Strain CBA and Sub-lines A, B and C of Strain NBT. The last 2 generations belonged entirely to cross Q × A. In $F_{12}$, which had the greatest number of affected mice, the incidences of 4·8 per cent in the females and of 11·9 per cent in the males were not statistically significantly different, and the total incidence in $F_{12}$ was 7·4 per cent.

Table VII shows the incidence of glandular stomach lesions in the NC group. There were 16 cases in 520 females (3·1 per cent) and 9 in 406 males (2·2 per cent), but the difference was not significant and the total incidence was 2·7 per cent. Although many unaffected mice lived longer than the oldest affected mouse, the average age at death of the former was less than that of affected mice in all

TABLE VIII.—*Data for Lesions in Glandular Stomach in Methylcholanthrene-injected M/CN Group of Hybrids. Earliest Lesion at 6·5 Months.*

| Generation and sex. | Effective number of mice. | Affected mice. No. | Affected mice. Age at death (months). Range. | Affected mice. Age at death (months). Average. | | Unaffected mice. Age at death (months). Range. | Unaffected mice. Age at death (months). Average. | | Type of cross. |
|---|---|---|---|---|---|---|---|---|---|
| $F_1$ F | 27 | 0 | — | — | — | 8·0–28·0 | 18·6 | 17·9 | — |
| M | 28 | 0 | — | — | | 8·0–28·0 | 17·1 | | |
| $F_2$ F | 74 | 0 | — | — | — | 8·0–28·0 | 15·5 | 14·3 | — |
| M | 60 | 0 | — | — | | 8·0–27·0 | 12·8 | | |
| $F_3$ F | 44 | 0 | — | — | 19·5 | 6.0–27·0 | 15·3 | 13·6 | QB |
| M | 45 | 1 | 19·5 | 19·5 | | 6·0–28·0 | 11·9 | | |
| $F_4$ F | 54 | 1 | 17·0 | 17·0 | 14·8 | 6·0–26·0 | 10·8 | 10·4 | PC |
| M | 47 | 1 | 12·5 | 12·5 | | 6·0–24·0 | 9·9 | | QB |
| $F_5$ F | 52 | 2 | 9·0–17·0 | 13·0 | 12·2 | 6·0–24·0 | 13·6 | 9·2 | PC |
| M | 56 | 1 | 10·5 | 10·5 | | 6·0–26·0 | 10·8 | | |
| $F_6$ F | 11 | 0 | — | — | — | 7·0–20·0 | 12·9 | 10·8 | — |
| M | 6 | 0 | — | — | | 6·0– 8·0 | 6·8 | | |
| $F_7$ F | 23 | 0 | — | — | — | 6·0–17·0 | 9·7 | 9·5 | — |
| M | 20 | 0 | — | — | | 6·0–16·0 | 9·3 | | |
| $F_8$ F | 44 | 0 | — | — | — | 6·0–18·0 | 10·3 | 9·1 | — |
| M | 56 | 0 | — | — | | 6·0–17·0 | 8·2 | | |
| $F_9$ F | 35 | 0 | — | — | 6·5 | 6·0–18·0 | 10·3 | 9·6 | PC |
| M | 31 | 1 | 6·5 | 6·5 | | 6·0–16·0 | 8·7 | | |
| $F_{10}$ F | 50 | 0 | — | — | 10·5 | 6·0·17·0 | 9·9 | 10·0 | PC |
| M | 36 | 1 | 10·5 | 10·5 | | 6·0–15·0 | 10·0 | | |
| Total F | 414 | 3 | 9·0–17·0 | 14·0 | 12·7 | 6·0–28·0 | 12·0 | 11·4 | — |
| M | 385 | 5 | 6·5–19·5 | 11·9 | | 6·0–28·0 | 10·8 | | |

generations except $F_8$. As shown in the last column of Table VII, the lesions occurred in the descendants of several types of cross between the original sub-lines.

The $F_1$ incidences were no higher than the incidences in later generations of the CN and NC hybrids.

The incidences in the two groups of reciprocal hybrids were compared and there was no significant difference ($\chi^2 = 3{\cdot}414$, $P > 0{\cdot}05$).

*The incidence of glandular gastric lesions in the injected reciprocal hybrids, M/CN and M/NC*

Tables VIII and IX show the incidences in each generation of the injected reciprocal hybrids. In the M/CN group there were 3 cases in 414 females (0·7 per cent) and 5 in 385 males (1·3 per cent); the difference between the incidences in the two sexes was not significant and the total incidence was 8 in 799 mice (1·0 per cent).

In the M/NC group there were 6 cases in 528 females (1·1 per cent) and 5 in 597 males (0·8 per cent); again the difference between the incidences in the two sexes was not significant and the total incidence was 11 in 1125 mice (1·0 per cent).

The incidences of the lesions in the two groups were therefore the same.

TABLE IX.—*Data for Lesions in Glandular Stomach in Methylcholanthrene-injected M/NC Group of Hybrids. Earliest Lesion at* 6 *Months.*

| Generation and sex. | Effective number of mice. | Affected mice. No. | Affected mice. Age at death (months). Range. | Average. | | Unaffected mice. Age at death (months). Range. | Average. | | Type of cross. |
|---|---|---|---|---|---|---|---|---|---|
| $F_1$ F | 17 | 0 | — | — | — | 9·0–31·0 | 17·6 | 17·6 | — |
| M | 23 | 0 | — | — | | 9·0–32·0 | 17·6 | | |
| $F_2$ F | 94 | 0 | — | — | 7·0 | 7·0–26·0 | 12·9 | 12·6 | BR |
| M | 97 | 1 | 7·0 | 7·0 | | 7·0–24·0 | 12·2 | | |
| $F_3$ F | 27 | 0 | — | — | — | 6·0–23·0 | 11·9 | 10·0 | — |
| M | 32 | 0 | — | — | | 6·0–13·0 | 8·4 | | |
| $F_4$ F | 94 | 1 | 12·0 | 12·0 | 12·0 | 6·0–29·0 | 12·1 | 10·7 | CP |
| M | 89 | 0 | — | — | | 6·0–24·0 | 9·3 | | |
| $F_5$ F | 48 | 0 | — | — | — | 6·0–24·0 | 9·7 | 9·1 | — |
| M | 64 | 0 | — | — | | 6·0–20·0 | 8·6 | | |
| $F_6$ F | 40 | 2 | 6·0–23·5 | 14·8 | 12·7 | 6·0–23·0 | 9·3 | 9·0 | CP |
| M | 40 | 1 | 8·0 | 8·0 | | 6·0–17·0 | 8·6 | | |
| $F_7$ F | 77 | 1 | 19·0 | 19·0 | 19·0 | 6·0–28·0 | 10·6 | 10·0 | CP |
| M | 79 | 0 | — | — | | 6·0–20·0 | 9·3 | | |
| $F_8$ F | 24 | 0 | — | — | — | 6·0–24·0 | 12·7 | 9·9 | — |
| M | 40 | 0 | — | — | | 6·0–17·0 | 8·3 | | |
| $F_9$ F | 48 | 1 | 18·0 | 18·0 | 18·0 | 6·0–23·0 | 10·7 | 10·5 | CP |
| M | 65 | 0 | — | — | | 6·0–22·0 | 10·4 | | |
| $F_{10}$ F | 59 | 1 | 18·0 | 18·0 | 18·5 | 6·0–27·0 | 10·7 | 10·7 | CP |
| M | 68 | 3 | 17·0–21·0 | 18·7 | | 6·0–25·0 | 10·7 | | |
| Total F | 528 | 6 | 6·0–23·5 | 16·0 | 15·2 | 6·0–31·0 | 11·5 | 10·8 | — |
| M | 597 | 5 | 7·0–21·0 | 14·2 | | 6·0–32·0 | 10·1 | | |

*Comparison of control and injected reciprocal hybrids*

The ages at death of the control and injected hybrids were compared. The lesions were found much earlier in the latter, the earliest being 6 months, compared with 14 months in the CN and 12 months in the NC group. The average age of affected mice was 9 months less in the M/CN than in the CN group, and 8 months less in the M/NC than in the NC group. But the injected mice had much shorter lives, on the whole, than the controls, the average age at death of unaffected injected mice being less than 12 months. In most generations the unaffected injected mice died at an earlier average age than the affected injected mice.

The incidences in the control groups were in both cases significantly higher than in the injected (NC *v.* M/NC, d = 1·7, 2 × SE = 1·2 ; CN *v.* M/CN, d = 3·4, 2 × SE = 1·6).

It will be seen from Table II that most of the lesions in the injected mice were classed as hyperplasia. This suggests that the pathological condition may begin at quite an early age and that, as it is only rarely lethal (by mechanical obstruction), it is found in a more advanced stage in mice dying in old age from other causes. There is therefore no absolute proof that, if the methylcholanthrene treatment was in any way connected with the development of the lesion, the carcinogen induced it earlier, but it seems possible. As the lesion is primarily one of old age, the much lower incidence in the injected mice was to be expected in mice dying young.

TABLE X.—*Data for Lesions in Glandular Stomach and Intestine in M/CN Uninjected Group. Earliest Lesion at* 10·5 *Months.*

| Generation and sex. | | Effective number of mice. | Affected mice. No. | Affected mice. Age at death (months). Range. | Average. | | Unaffected mice. Age at death (months). Range. | Average. | | Type of cross. |
|---|---|---|---|---|---|---|---|---|---|---|
| $F_5$ | F | 38 | 1 | 23·5 | 23·5 | 23·5 | 11·0–25·0 | 19·3 | 18·7 | PC |
| | M | 49 | 0 | — | — | | 10·0–25·0 | 18·3 | | |
| $F_6$ | F | 3 | 0 | — | — | — | 16·0–20·0 | 18·0 | 17·8 | — |
| | M | 2 | 0 | — | — | | 14·0–21·0 | 17·5 | | |
| $F_7$ | F | 17 | 4* | 13·0–17·0 | 15·4 | 15·4 | 10·0–18·0 | 15·5 | 14·9 | PC |
| | M | 24 | 0 | — | — | | 12·0–17·0 | 14·6 | | |
| $F_8$ | F | 31 | 0 | — | — | 10·5 | 11·0–19·0 | 15·4 | 14·3 | PC |
| | M | 38 | 1 | 10·5 | 10·5 | | 10·0–17·0 | 13·3 | | |
| $F_9$ | F | 40 | 0 | — | — | — | 11·0–21·0 | 16·3 | 15·6 | — |
| | M | 36 | 0 | — | — | | 10·0–22·0 | 14·9 | | |
| $F_{10}$ | F | 46 | 0 | — | — | — | 10·0–19·0 | 14·7 | 14·0 | — |
| | M | 38 | 0 | — | — | | 10·0–20·0 | 13·1 | | |
| $F_{11}$ | F | 90 | 1 | 19·5 | 19·5 | 19·5 | 10·0–24·0 | 13·5 | 12·4 | PC |
| | M | 85 | 0 | — | — | | 10·0–21·0 | 11·2 | | |
| $F_{12}$ | F | 114 | 0 | — | — | — | 10·0–20·0 | 13·6 | 13·3 | — |
| | M | 125 | 0 | — | — | | 10·0–17·0 | 13·1 | | |
| Total | F | 380 | 6 | 13·0–23·5 | 17·4 | 16·4 | 10·0–25·0 | 14·8 | 14·2 | — |
| | M | 397 | 1 | 10·5 | 10·5 | | 10·0–25·0 | 13·6 | | |

* 1 mouse (No. 38) had stomach lesion only; 1 mouse (No. 39) had duodenal lesion only; 2 mice (Nos. 34 and 37) had stomach and intestinal lesions.

TABLE XI.—*Data for Lesions in Glandular Stomach and Intestine in M/NC Uninjected Group. Earliest Lesion at* 15 *Months.*

| Generation and sex. | Effective number of mice. | Affected mice. No. | Affected mice. Age at death (months). Range. | Affected mice. Age at death (months). Average. | | Unaffected mice. Age at death (months). Range. | Unaffected mice. Age at death (months). Average. | | Type of cross |
|---|---|---|---|---|---|---|---|---|---|
| $F_5$ F | 19 | 0 | — | — | — | 16·0–27·0 | 22·8 | 21·8 | — |
| M | 19 | 0 | — | — | | 15·0–29·0 | 20·8 | | |
| $F_6$ F | 90 | 8* | 20·0–26·5 | 22·6 | 23·0 | 15·0–29·0 | 22·6 | 20·1 | CP |
| M | 90 | 4† | 21·0–24·5 | 23·6 | | 15·0–30·0 | 17·7 | | |
| $F_7$ F | 68 | 10 | 18·5–29·0 | 24·8 | 23·7 | 16·0–28·0 | 22·2 | 20·5 | CP |
| M | 59 | 4† | 17·0–26·5 | 20·9 | | 15·0–27·0 | 18·8 | | |
| $F_8$ F | 37 | 1 | 23·5 | 23·5 | 23·5 | 16·0–28·0 | 23·2 | 21·2 | CP |
| M | 24 | 0 | — | — | | 15·0–26·0 | 18·1 | | |
| $F_9$ F | 32 | 2 | 19·0–24·0 | 21·5 | 21·2 | 15·0–28·0 | 21·0 | 19·8 | CP |
| M | 33 | 1 | 20·5 | 20·5 | | 15·0–26·0 | 18·6 | | |
| $F_{10}$ F | 68 | 3 | 23·5–26·0 | 25·2 | 23·1 | 15·0–28·0 | 21·6 | 21·0 | CP |
| M | 62 | 5 | 19·0–25·0 | 21·8 | | 15·0–27·0 | 20·3 | | |
| $F_{11}$ F | 107 | 7 | 21·0–25·5 | 23·7 | 22·4 | 15·0–28·0 | 20·6 | 19·6 | CP |
| M | 155 | 10 | 17·0–23·0 | 21·6 | | 15·0–25·0 | 18·9 | | |
| $F_{12}$ F | 284 | 18 | 17·0–26·5 | 22·6 | 22·1 | 15·0–28·0 | 21·3 | 21·0 | CP |
| M | 366 | 35 | 15·0–28·5 | 21·8 | | 15·0–28·0 | 20·9 | | |
| Total F | 705 | 49 | 17·0–29·0 | 23·4 | 22·5 | 15·0–29·0 | 21·6 | 20·6 | — |
| M | 808 | 59 | 15·0–28·5 | 21·8 | | 15·0–30·0 | 19·8 | | |

* Includes mouse with tumours of rectum and colon.
† One duodenal lesion included in each case.

Although there were more instances of affected mice in the $F_1$ controls than in the $F_1$ injected mice (there were no cases in the latter), the fact that the incidence in the $F_1$ controls was no higher than in the later generations of controls (where many of the mice were equally long-lived) suggests that the possible contamination of some $F_1$ mice by methylcholanthrene from their injected cage-mates did not affect the incidence of glandular gastric lesions. Previously it has been shown that this contamination raised the incidence of lung tumours but did not affect the incidence of forestomach papillomata (Miller and Pybus, 1954*b*, 1955).

As the injected mice of both groups were derived mainly from crosses between Sub-lines C (NBT) and P (CBA) most of the lesions appeared in descendants of these crosses, but a few occurred in the descendants of crosses between the other sub-lines.

*The incidence of glandular gastric and intestinal lesions in the uninjected descendants of injected hybrids.*

Tables X and XI give the relevant data for $F_5$ to $F_{12}$ of the M/CN and M/NC *uninjected* groups.

Many of the uninjected M/CN mice died young, as shown in Table X. Consequently there were few mice with lesions of the glandular stomach. There were 7 affected mice (6 females and 1 male) in a total of 777 mice (0·9 per cent). The incidence in females of 6 in 380 (1·6 per cent) was not significantly different from that in males of 1 in 397 (0·25 per cent) (d = 1·328, 2SE = 1·372). All the lesions occurred in the descendants of crosses between Sub-lines P and C. In $F_7$, of 4 affected females, 2 had duodenal or lower intestinal lesions in addition to those in the stomach, and one had a duodenal adenoma only.

The greatest number of lesions occurred in the uninjected M/NC group. These mice were much longer-lived and instances of the lesions were seen in all generations except $F_5$. There was no significant sex-difference in incidence ; 49 females in 705 (7·0 per cent) and 59 males in 808 (7·3 per cent) were affected, a total of 108 mice in 1513 (7·1 per cent), the earliest case appearing at 15 months. There were 3 duodenal tumours, 2 in $F_6$ and 1 in $F_7$. As shown in Table XI, the average age at death of unaffected mice was less in every generation than that of affected mice by as much as 2 to 3 months.

The incidence in the M/NC uninjected mice was significantly higher than that in the M/CN uninjected group (d = 6·2, 2SE = 1·5). Comparison of Tables X and XI shows that the M/CN uninjected mice died on an average 6 months earlier and this affected the incidence not only of gastric lesions but also of all types of tumours in this group.

All the lesions in these two groups appeared in the descendants of crosses between Sub-lines C (of NBT) and P (of CBA).

*Comparison of the various hybrid groups*

The incidence of gastric lesions in the M/CN uninjected group was not significantly different from that in the M/CN injected group ; the uninjected mice lived a little longer, but both died early.

The incidence in the CN group was significantly higher than that in the M/CN uninjected group (d = 3·4, 2SE = 1·6), and the difference in age was sufficient to account for this, the uninjected M/CN unaffected mice dying 7 months earlier than the CN unaffected mice.

The incidence in the M/NC uninjected group was significantly higher than that in the injected group (d = 6·1, 2SE = 1·4), a difference which might be accounted for by the longer life of the uninjected mice ; unaffected mice of the uninjected group lived on an average 10 months longer than those of the injected group. But the incidence in the uninjected group was also significantly higher than that in the NC mice (d = 4·4, 2SE = 1·7). Although the average ages at death for all generations in the two groups were very similar, a comparison of the generations from $F_5$ to $F_{12}$ showed that the average age at death of the uninjected M/NC mice was 2 to 3 months greater (5·5 months in $F_{12}$). The complete absence of lesions from the later generations of NC mice might be due partly to the shorter lives of these animals compared with the earlier generations.

The figures in Table XI are based on the numbers alive at 15 months, while those in Table VII are based on the numbers alive at 12 months, these being the ages at which the earliest lesion in each group occurred. The figures for the uninjected M/NC group were revised on a basis of 12 months, for strict comparison, but the total average age at death was reduced by only 1 month from 20·6 months to 19·8, with corresponding reductions in each generation, so that they were now from 1 to 2 months greater (and 4·8 months in $F_{12}$) ; the difference in incidence between the M/NC uninjected group and the NC group was still significant (d =

3·8, 2SE = 1·6), there being 49 cases in the new total of 761 females (6·4 per cent) and 59 in 913 males (6·5 per cent), giving a total incidence of 6·5 per cent.

*Heredity*

While the longevity of the animals undoubtedly plays a large part in the incidence of tumours in a strain, especially those tumours which appear mainly in old mice, there is also the question of heredity.

The incidence of glandular stomach lesions in the parent NBT strain (4·2 per cent) was not significantly different from the total incidence in the reciprocal hybrids (4·3 per cent in CN, 2·7 per cent in NC), nor from that in the M/NC uninjected group (7·1 per cent). But the incidence in the parent CBA strain (0·2 per cent), while not differing significantly from that of NBT owing to the small numbers of the latter, was significantly less than that of the CN group (d = 4·1, 2SE = 1·6) and that of the NC group (d = 2·5, 2SE = 1·2) and that of the M/NC uninjected group (by inspection), all of which consisted of large numbers of mice.

It is concluded that the hybrids inherited the character from the NBT strain, and that the crossing of the two strains of itself did not significantly increase the incidence; the CN $F_1$ incidence was 4 in 106 mice (3·8 per cent), and the NC $F_1$ incidence was 2 in 95 mice (2·1 per cent).

The character was present in the NBT strain in Sub-line C, but appeared in the hybrids in crosses involving also Sub-lines A and B. The fact that the character appeared once in the CBA strain controls during the experiment, and once again later on, suggests that it is a character which might be due to an unstable gene liable to spontaneous mutation. The second CBA case appeared in Sub-line R, ln a later generation than those used in the experiment.

TABLE XII.—*The Incidence of Lesions of Glandular Stomach and Intestine in Those Families of the M/NC Uninjected Group to which Affected Mice in Each Generation Belonged.*

| Generation. | Sex. | Effective number of mice. | Affected mice. Number. | Affected mice. Percentage. | | Number of affected families. |
|---|---|---|---|---|---|---|
| $F_6$ | F | 33 | 8 | 24·2 | 17·1 | 6 |
| | M | 37 | 4 | 10·8 | | |
| $F_7$ | F | 25 | 10 | 40·0 | 32·6 | 6 |
| | M | 21 | 4 | 19·1 | | |
| $F_8$ | F | 3 | 1 | 33·3 | 25·0 | 1 |
| | M | 1 | 0 | 0·0 | | |
| $F_9$ | F | 9 | 2 | 22·2 | 15·8 | 3 |
| | M | 10 | 1 | 10·0 | | |
| $F_{10}$ | F | 30 | 3 | 10·0 | 15·7 | 7 |
| | M | 21 | 5 | 23·8 | | |
| $F_{11}$ | F | 43 | 7 | 16·3 | 16·2 | 11 |
| | M | 62 | 10 | 16·1 | | |
| $F_{12}$ | F | 151 | 18 | 11·9 | 14·6 | 31 |
| | M | 213 | 35 | 16·4 | | |
| Total | F | 294 | 49 | 16·7 | 16·4 | — |
| | M | 365 | 59 | 16·2 | | |

Although the general incidence in the hybrids was no higher than in the NBT mice, the greatest number of lesions occurred in the M/NC uninjected group. An analysis was made of only those families, in each generation of the latter group, in which affected mice were found, as shown in Table XII. The differences between the sexes were not significant in any generation. The incidence in $F_7$ was significantly higher than that in $F_{11}$ (d = 16·4, 2SE = 15·6) and that in $F_{12}$ (d = 18·0, 2SE = 14·3), but the differences between the other generations were statistically insignificant. There is here no indication that the incidence of the lesions was increasing in later generations after injection of the preceding generations with methylcholanthrene.

An attempt was made, by studying the pedigree charts of all the mice in the experiment, to arrive at some conclusion as to the mode of inheritance of the character. Strong (1945) found that in his mice the character was inherited as a dominant and depended on a single major gene carried by the " brown-tagged " chromosome. Table XIII shows the distribution in the present experiment of the stomach and intestinal lesions according to coat colour. It will be seen that 119 of the 196 cases occurred in brown mice. Except in the M/NC uninjected group, where all the affected mice bred from were either brown agouti or white, only 10 affected mice were bred from, and 5 of these were brown (agouti or non-agouti) while the other 5 were black (agouti or non-agouti). By their breeding analysis 4 of these latter 5 were heterozygous for brown ; the fifth case was a

TABLE XIII.—*The Classification According to Coat Colour of Mice with Glandular Stomach and Intestinal Lesions in the Various Groups of Two Reciprocal-hybrid Strains.*

| | Group of mice. | | | | | | |
|---|---|---|---|---|---|---|---|
| Coat colour. | CN. | M/CN injected. | M/CN uninjected. | NC. | M/NC injected. | M/NC uninjected. | Total. |
| Black agouti (CBA) | 8 | 4 | 6 | 12 | 1 | 0 | 31 |
| Brown agouti (CbA) | 20 | 1 | 0 | 3 | 5 | 84 | 113 |
| Black non-agouti (CBa) | 1 | 0 | 0 | 5 | 0 | 0 | 6 |
| Brown non-agouti (Cba) | 4 | 0 | 0 | 2 | 0 | 0 | 6 |
| White (cc) | 4 | 3 | 1 | 3 | 5 | 24 | 40 |
| Total | 37 | 8 | 7 | 25 | 11 | 108 | 196 |

black mouse (NC $F_5$/163), the offspring of 2 generations of black mice, and, mated with its black brother, it gave 7 generations of only black mice, including 2 in $F_7$ with stomach lesions. A black mouse, heterozygous for brown, mated with a homozygous black would produce only black mice, and while it is perhaps straining the bounds of coincidence for this type of mating to take place in 8 successive generations, there is the possibility that these 3 mice were heterozygous for brown. On the other hand, a few lesions of the glandular stomach mucosa, apparently of the type described here, have been found in old C57BL mice (Andervont, 1939).

There was therefore evidence from the present experiment to support Strong (1945) in his statement that the factor for susceptibility is carried on the " brown " chromosome.

Further examination of the pedigree charts provided evidence that the character was not inherited as a dominant.

In the CN group, cases occurred in the descendants of NBT males 10075 and 10076 (Sub-line B), 10047 and 10049 (Sub-line C) and 10068 (Sub-line A), crossed with CBA mice of the Sub-lines Q, P and Q respectively. One example, in the descendants of the QB cross, is shown in Fig. 10 ; in one line (which finished in $F_7$) 2 affected males were bred from in $F_3$ and $F_4$—in $F_4$ the incidence was 1 in 8 mice, in $F_5$ it was 1 in 14, this latter mouse having a duodenal lesion ; in the other line (shown as far as $F_7$) there was one case in $F_5$ and none in later generations, although there were 50 mice in $F_8$ to $F_{12}$.

In the reciprocal NC hybrids, affected mice occurred amongst the offspring of NBT females 10066 and 10067 (Sub-line A), 10073 and 10074 (Sub-line B), 10044 and 10046 (Sub-line C), and 10085 and 10086 (Sub-line D). Fig. 11 shows the descendants of Sub-line B crossed with Sub-line R of Strain CBA ; there were no affected mice after $F_4$, although in $F_5$ to $F_{12}$ there were 94 mice from 10074 and 110 from 10073.

In the M/CN (injected) group, 2 cases occurred in the descendants of NBT males 10075 and 10076 (Sub-line B), and 6 in the descendants of NBT male 10048 (Sub-line C) ; these 6 were all descended from one $F_2$ pair, 322 (female) × 327 (male).

All 7 cases occurring in the M/CN (uninjected) group belonged to the P × C cross and were descended from this same $F_2$ pair 322 × 327. An interesting feature about this group was that intestinal lesions appeared in 2 $F_7$ litters which

---

EXPLANATION OF PLATES

Fig. 1.—Pedunculate adenoma of stomach, with early penetration of muscularis mucosae at apex of stalk ; one of two polypoid adenomata in same mouse, NC $F_4$/136, 22 months old female. × 25.

Fig. 2.—Part of a sessile adenoma near pylorus showing penetration of muscularis mucosae at one point, with development of large epithelium-lined cyst in submucosa ; glandular arrangement of epithelium within wall of cyst and polypoid projection into lumen. Mouse CN $F_{12}$/454, 14·5 months old female. × 75.

Fig. 3.—Adenomatous alteration of Brunner's gland beneath stomach adenoma ; note scirrhous nature of adenoma. Mouse CN $F_3$/97, 26 months old female. × 75.

Fig. 4.—Part of pedunculate adenocarcinoma of stomach with penetration and disruption of muscularis mucosae at apex of stalk. Epithelium-lined cyst passing out through circular muscle in track of blood vessels. Mouse M/NC $F_{12}$/13, uninjected 21 months old female. × 75.

Fig. 5.—Adenocarcinoma of stomach, showing disruption of muscularis mucosae and submucosal tissues at base of tumour, and passage of epithelium-lined cyst through circular muscle. Mouse M/NC $F_7$/407, uninjected 28 months old female. × 16.

Fig. 6.—Oxyntic-cell carcinoma, passing through and disrupting all layers of muscle and projecting beyond outer wall of stomach. Mouse M/NC $F_{10}$/297, uninjected 26 months old female. × 16.

Fig. 7.—Enlargement of adjacent section to Fig. 6, towards inner surface of tumour, showing strand of muscle within tumour, glandular cysts containing polymorphs, and groundwork of acidophilic (oxyntic) cells. × 67.

Fig. 8.—Part of duodenal adenoma, 3 mm. in length. Disorderly atypical epithelium dipping down into apparently normal Brunner's glands, disorganizing the muscularis mucosae. Mouse M/CN $F_7$/34, uninjected 16·5 months old female. × 22.

Fig. 9.—Adenoma of rectum. Point of penetration of muscularis mucosae not seen in photograph ; gross infiltration of leucocytes into apex of stalk seen in upper part of photograph. Mouse M/NC $F_6$/70, uninjected 20 months old female. × 22.

1

2

3

4

Miller and Pybus.

5

6

7

8

9

came from matings of 2 injected $F_6$ females with their injected brother ; in one litter of 4 females and 4 males there were 3 affected females (one with stomach lesion only, one with duodenal lesion only and one with stomach lesion and intestinal polyp) and in the other litter of 3 females and 10 males there was one female with stomach and duodenal lesions. Unfortunately the NBT male 10048, from which apparently the character was inherited in the PC cross, was bred from in the control CN group to $F_2$ only and no stomach or intestinal lesions were seen in its 34 descendants in that group.

FIG. 10.—Part of CN pedigree, showing occurrence of spontaneous stomach and intestinal lesions in two lines of descendants from the cross between Sub-line Q of Strain CBA and Sub-line B of Strain NBT. For explanation of symbols see Fig. 11.

In the M/NC injected group, 10 of the 11 cases occurred amongst the descendants of NBT female 10046 (Sub-line C) and all were descended from one $F_2$ pair 322 (female) × 319 (male). (It was a pure coincidence that in the reciprocal M/NC and M/CN groups the 2 $F_2$ female ancestors of all the affected mice should bear the same number, 322.) From M/NC $F_2$ 322 × 319, 7 $F_3$ pairs were bred from ; 4 of these produced no affected offspring in 104 descendants to $F_{10}$ ; 2 of the pairs had one case each, one in $F_4$ and one in $F_6$, the lines being discontinued at these

FIG. 11.—Part of NC pedigree, showing occurrence of spontaneous stomach lesions in descendants from two crosses between Sub-line B of Strain NBT and Sub-line R of Strain CBA.

FIG. 12.—Part of M/NC pedigree, showing occurrence of spontaneous stomach and intestinal lesions in the uninjected descendants of injected mice in the cross between Sub-line C of Strain NBT and Sub-line P of Strain CBA. All mice injected up to $F_5$ ; $F_6$ litters from mice No. 176 × No. 182, and from mice No. 86 × No. 90 were injected. All affected mice from $F_8$ onwards in this group were descended from $F_6$ mice No. 57 × No. 59.

generations respectively ; the fifth pair produced the other 8 cases, 2 (brother and sister) in $F_6$, 1 in $F_7$, 1 in $F_9$ and 4 in $F_{10}$. The last 5 cases came from the same $F_6$ pair, from 2 $F_7$ pairs, and from 4 $F_8$ pairs. If a mutation is to be assumed to be the cause of the sporadic appearance of the character, then either it took place not later than in the $F_2$ mice 322 or 319, or else it occurred several times in different mice in $F_3$ to $F_8$ ; and $F_2$ was so close to the parent strains that the possibility of inheritance from them cannot be discarded in favour of mutation.

This is illustrated more clearly in the M/NC uninjected group, of which a part is shown in Fig. 12. All the affected mice in this group were descended from the same pair of $F_2$ mice 322 $\times$ 319 which produced all the cases in the injected line. The chart shows all the affected mice up to $F_7$. In the later generations all affected mice were descended from the $F_6$ pair 57 $\times$ 59 ; they occurred singly or in twos per litter except for one litter in $F_{11}$, which had 4 cases in 13 mice, and 4 litters in $F_{12}$ each containing 3 cases (in families of 21, 6, 15 and 20 mice respectively), and one outstanding $F_{12}$ family where there were 8 affected mice (5 females, 3 males) in a total of 21 (14 females and 7 males). This last family had no affected mouse in its direct ancestry right back to $F_1$, but there was one affected $F_{10}$ male, the uninjected brother of its injected grandparents.

While demonstrating that a tendency to the condition appears to be inherited, this material gives no indication of the number of factors involved. In Strong's NHO strain (Strong, 1945) the character was shown to be inherited as a dominant, but Andervont (1939) found that the Strain I lesion was inherited as a recessive.

### DISCUSSION

This communication describes the incidence and histology of glandular lesions in the gastric and intestinal mucosa which closely resemble many of the cases described in the literature. The early stages of the Strain I lesion are very similar to some of the present examples, although the later stages are far more extensive and diffuse than the much more local changes seen in the present material. The early " precancerous " stages of the induced gastric lesions are identical with many of the present cases. Strong (1947) gave instances of more extensive metastasizing lesions, but later surveys of some of his material by McPeak and Warren (1947) and by Kaplan (1949) failed to show such malignant pictures ; from the last two descriptions it appears that the lesions obtained in the present material are identical with most of Strong's.

There has been much discussion in the literature as to the classification of these lesions, some of which has already been referred to in this communication in the description of the histology. A statement by Klein and Palmer (1941) seems to have special application to the present lesions. It is to the effect that, from experience, it is now possible to predict that a spontaneous tumour will follow a malignant course, on the basis of its structure only and without any proof of malignant activity beyond a few microscopic changes which, *if they did not progress* (our italics), could not be classified as malignant. Detailed study of the present lesions seems to show a succession of stages leading up to true malignancy but failing to reach it during the lifetime of the mouse. This idea of progression has been developed by Foulds (1949, 1950, 1951) and by Shubik, Baserga and Ritchie (1953). The successful induction of true gastric carcinoma (Stewart and Lorenz, 1941, 1942, 1949 ; Howes and Oliviera, 1948 ; Stewart, Hare,

Lorenz and Bennett, 1949 ; Stewart, Hare and Bennett, 1953) and of intestinal carcinoma (Lorenz and Stewart, 1940 ; Cox, Wilson and DeEds, 1947 ; Miller, Sandin, Miller and Rusch, 1955) shows that the progression can be speeded up by direct injection or by feeding of a carcinogen. Strong (1945, 1947) described the invasion of surrounding organs and the development of metastases in some of his mice, showing that in a suitably susceptible strain of mice true malignancy could be achieved in the lifetime of the mouse.

In the present material there was by definition one instance only of a true carcinoma and even that did not produce metastases.

There was slight evidence from this experiment that the treatment with methylcholanthrene might have speeded up the development of the lesions, thus confirming Kaplan's conclusions (Kaplan, 1949) ; a greater proportion of the earlier stages (hyperplasia) were seen in injected mice, at an earlier age. This might be explained by the fact that a greater number of these mice came early to autopsy. This fact made it impossible to show that the carcinogen had in any way intensified the incidence of the lesions in the injected mice, as a sufficient number of the latter failed to live to an age comparable with that of the controls. Deringer, Heston and Barrett (1953) found that methylcholanthrene did not increase the incidence of lesions in the glandular stomachs of ST mice.

Strong (1945, 1947) explained the increased incidence of the lesions in the NHO strain by assuming that the carcinogen had produced a heritable germinal mutation, but acknowledged the biologic variability of his mice due to hybridisation. A study of the pedigree charts in the present experiment has shown that if the methylcholanthrene induced a mutation it did so in $F_2$ in both the reciprocal crosses, as one pair of $F_2$ mice in each group of hybrids gave rise to all the affected $F_{12}$ mice ; or, alternatively, so many separate mutations must have been induced to account for the presence of affected mice in a large number of sub-lines that the number quite surpasses any ordinary known rate of mutation. It is believed that in this experiment the increased incidence of the lesions was due to hybridity and segregation of the character ; the character was present in both parent strains but especially in the NBT, and the charts proved that it was inherited, but not as a dominant. Different families and generations showed different incidences, but, within the limits of the experiment, there was no permanently increased incidence. Had it been possible to continue breeding after $F_{12}$, especially in the more susceptible lines, it seems likely that a line with a high spontaneous incidence might have become established.

Since this work was concluded the same character has appeared in the GFF/G strain ; in the first 7 inbred generations there were 7 cases in 85 females (8·2 per cent). These mice are brown with pink-eye dilution and further evidence is thus provided for the transmission of the character by the " brown " chromosome.

### SUMMARY

An account is given of the histology and incidence of localised lesions of the glandular part of the stomach and of the intestinal mucosa, which occurred spontaneously in older mice of two hybrid strains obtained by reciprocal crosses between the NBT and CBA strains, and inbred for twelve generations.

The incidence of the lesions was the same in the reciprocal hybrids and in the NBT strain. Two isolated instances were seen in the CBA strain.

The lesions were of a hyperplastic, adenomatous and adenocarcinomatous nature ; one true carcinoma of the stomach was seen. There were no metastases and no invasions of neighbouring organs.

The character was inherited, although not as a dominant, and appeared to be carried on the " brown " chromosome.

While the appearance of the early stages of development of the lesions might have been accelerated by subcutaneous methylcholanthrene injections into one group of each of the reciprocal hybrids, there was no evidence that the carcinogen had caused a germinal mutation or that the incidence was increased in the uninjected descendants of injected mice.

Some of the microscopical preparations have been examined by Dr. Ian Rannie, Lecturer in Pathology in King's College, University of Durham, and we wish to express our sincere thanks to him for his helpful advice. Our thanks are due also to Dr. U. Philip, Lecturer in Genetics in King's College, for her interest in the genetical aspect of the experiment.

The work was carried out under a research grant from the North of England Council of the British Empire Cancer Campaign.

### REFERENCES

ANDERVONT, H. B.—(1939) *Publ. Hlth Rep. Wash.*, **54**, 1851.—(1949) *J. nat. Cancer Inst.*, **10**, 405.
CHRISTIE, A. C.—(1953) *Brit. J. Cancer*, **7**, 65.
COX, A. J., WILSON, R. H. AND DEEDS, F.—(1947) *Cancer Res.*, **7**, 647.
DERINGER, M. K., HESTON, W. E. AND BARRETT, M. K.—(1953) *J. nat. Cancer Inst.*, **14**, 375.
FOULDS, L.—(1949) *Brit. J. Cancer*, **3**, 345.—(1950) *J. R. micr. Soc.*, **70**, 173.—(1951) *Ann. R. Coll. Surg. Engl.*, **9**, 93.
HOWES, E. L. AND OLIVIERA, J. ROZA DE.—(1948) *Cancer Res.*, **8**, 419.
KAPLAN, H. S.—(1949) *J. nat. Cancer Inst.*, **10**, 407.
KLEIN, A. J. AND PALMER, W. L.—(1941) *Ibid.*, **1**, 559.
LORENZ, E. AND STEWART, H. L.—(1940) *Ibid.*, **1**, 17.
McPEAK, E. AND WARREN, S.—(1947) *Ibid.*, **7**, 309.
MILLER, E. W. AND PYBUS, F. C.—(1945*a*) *Brit. J. Cancer*, **8**, 163.—(1954*b*) *Ibid.*, **8**, 466.—(1955) *Ibid.*, **9**, 142.
MILLER, J. A., SANDIN, R. B., MILLER, E. C. AND RUSCH, H. P.—(1955) *Cancer Res.*, **15**, 188.
SHUBIK, P., BASERGA, R. AND RITCHIE, A. C.—(1953) *British J. Cancer*, **7**, 342.
SMITH, F. W. AND STRONG, L. C.—(1949) *J. nat. Cancer Inst.*, **10**, 423.
STEWART, H. L.—(1940) *Arch. Path.*, **29**, 153.—(1941) *J. nat. Cancer Inst.*, **1**, 489.
*Idem* AND ANDERVONT, H. B.—(1938) *Arch. Path.*, **26**, 1009.
*Idem*, HARE, W. V. AND BENNETT, J. G.—(1953) *J. nat. Cancer Inst.*, **14**, 105.
*Idem*, HARE, W. V., LORENZ, E. AND BENNETT, J. G.—(1949) *Ibid.*, **10**, 359.
*Idem* AND LORENZ, E.—(1941) *Ibid.*, **2**, 193.—(1942) *Ibid.*, **3**, 175.—(1949) *Ibid.*, **10**, 147.
Strong, L. C.—(1945) *Ibid.*, **5**, 339.—(1947) *Ibid.*, 7, 305.
*Idem*, COLLINS, V. J. AND DURAND, E. A.—(1943) *Cancer Res.*, **3**, 21.


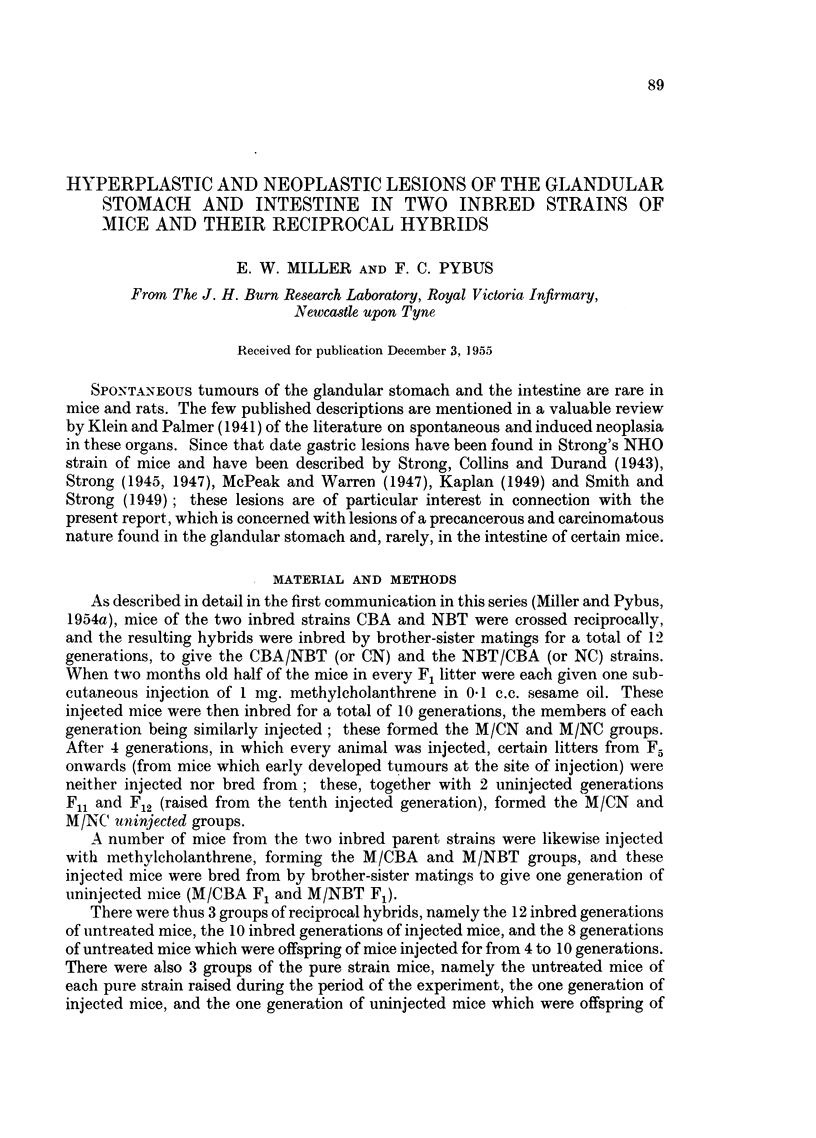

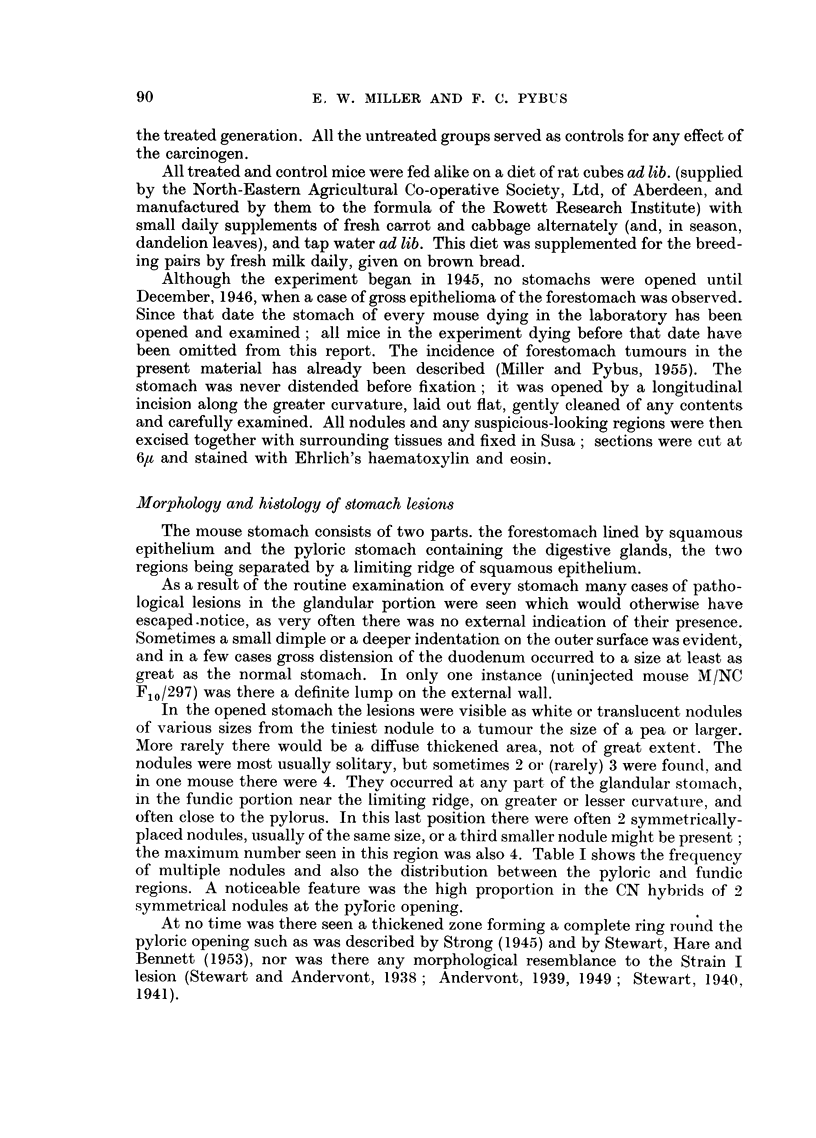

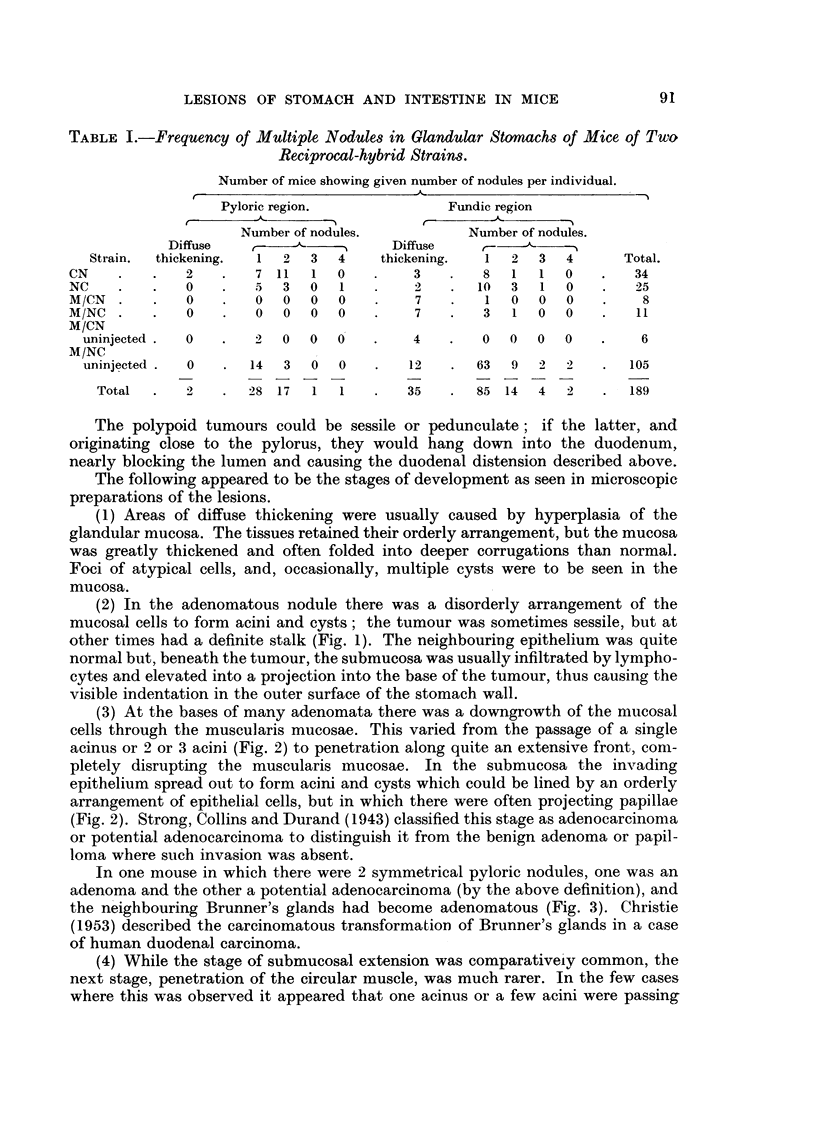

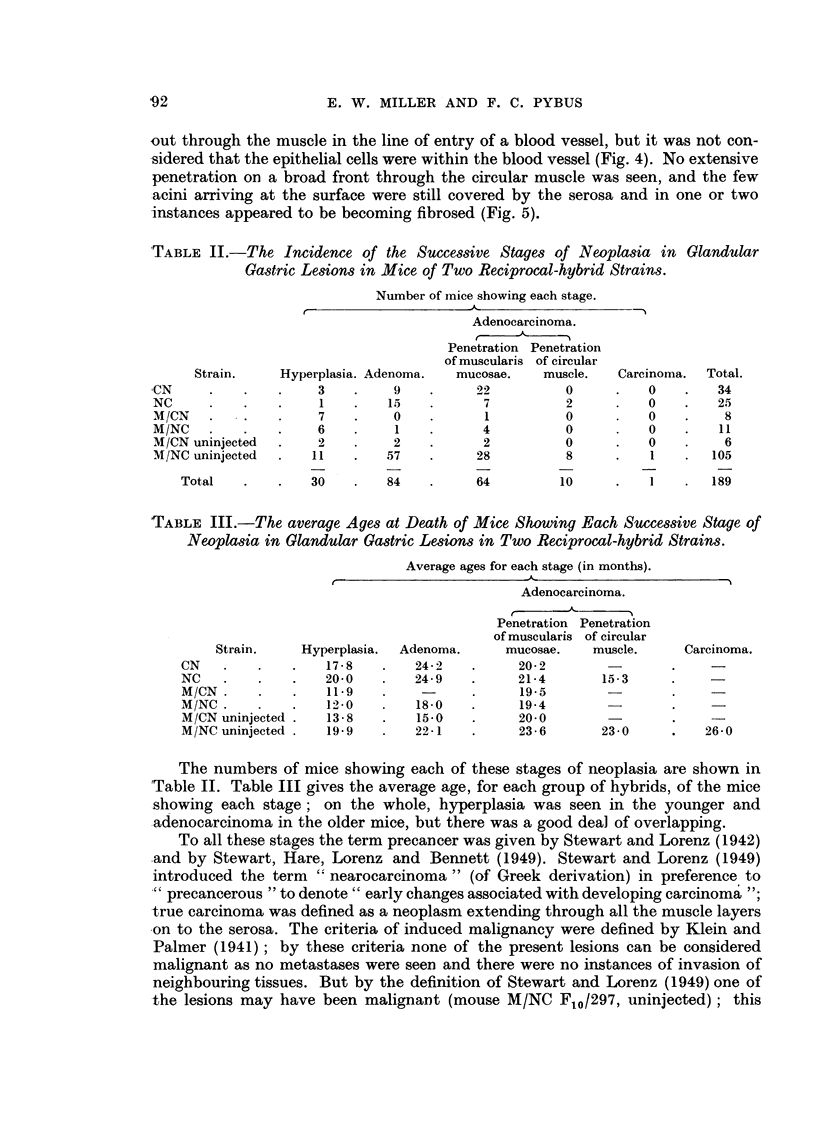

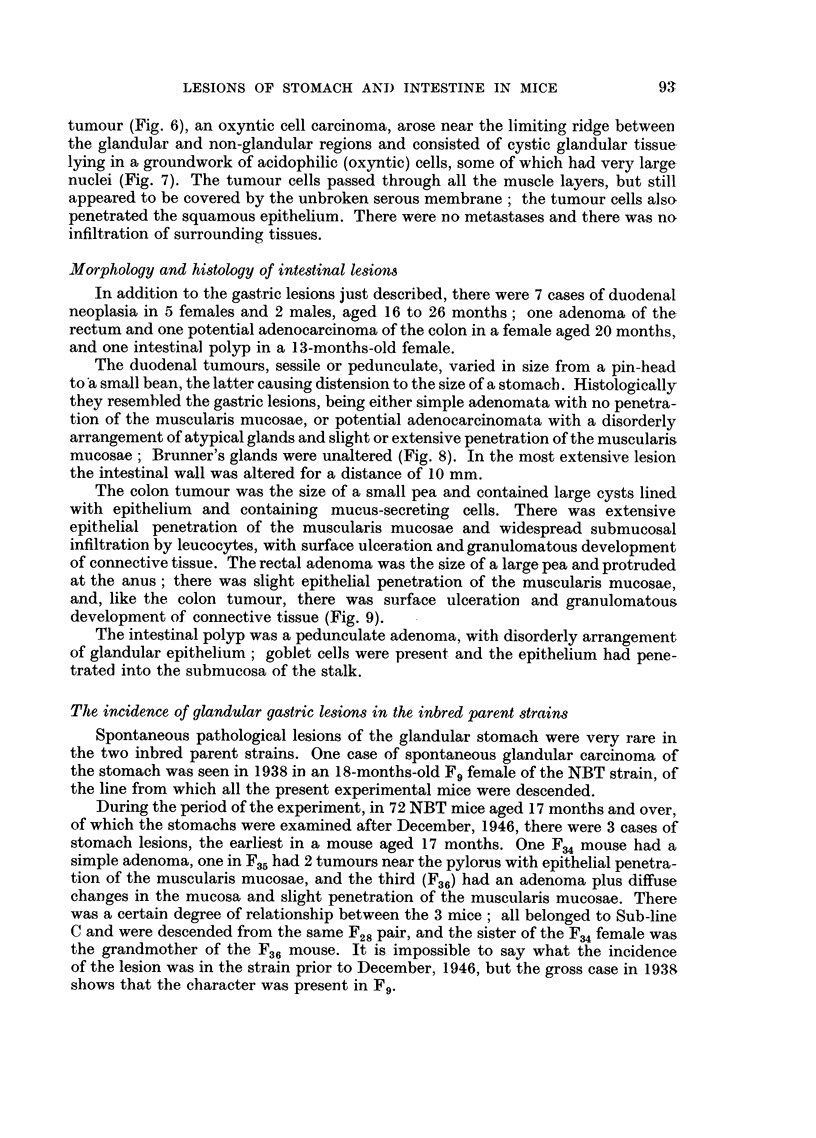

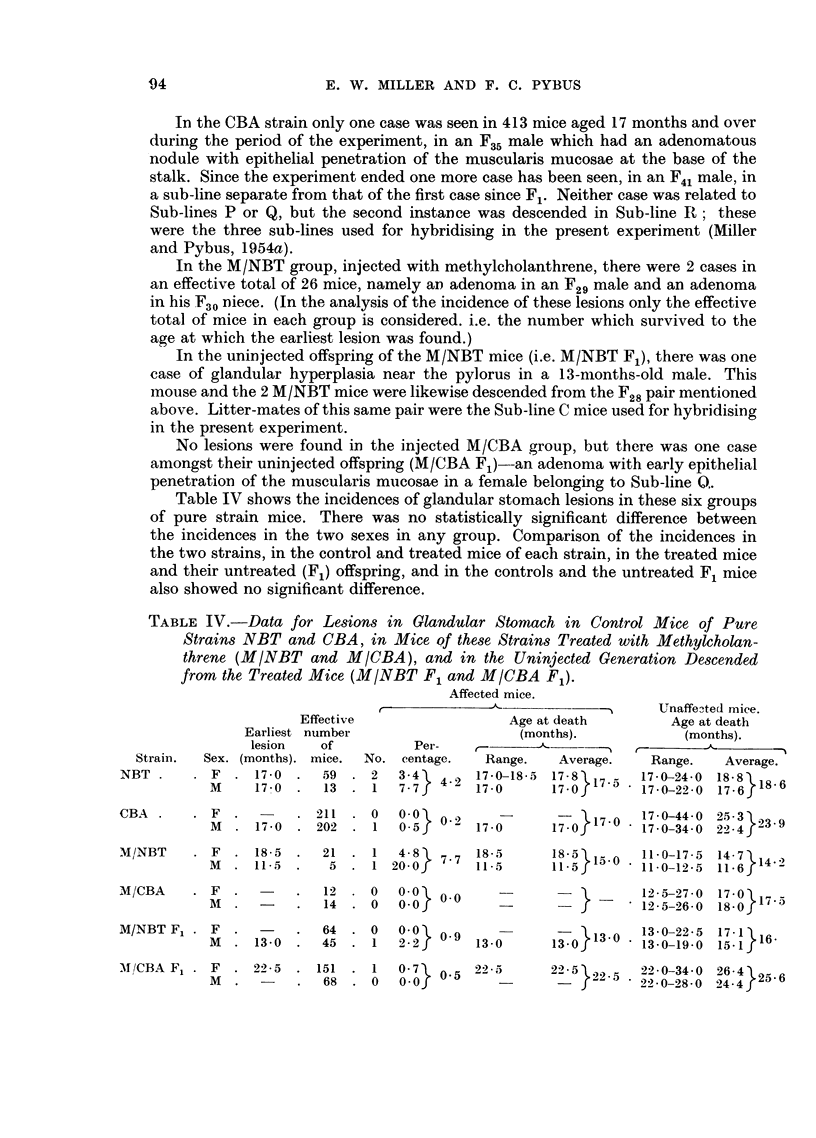

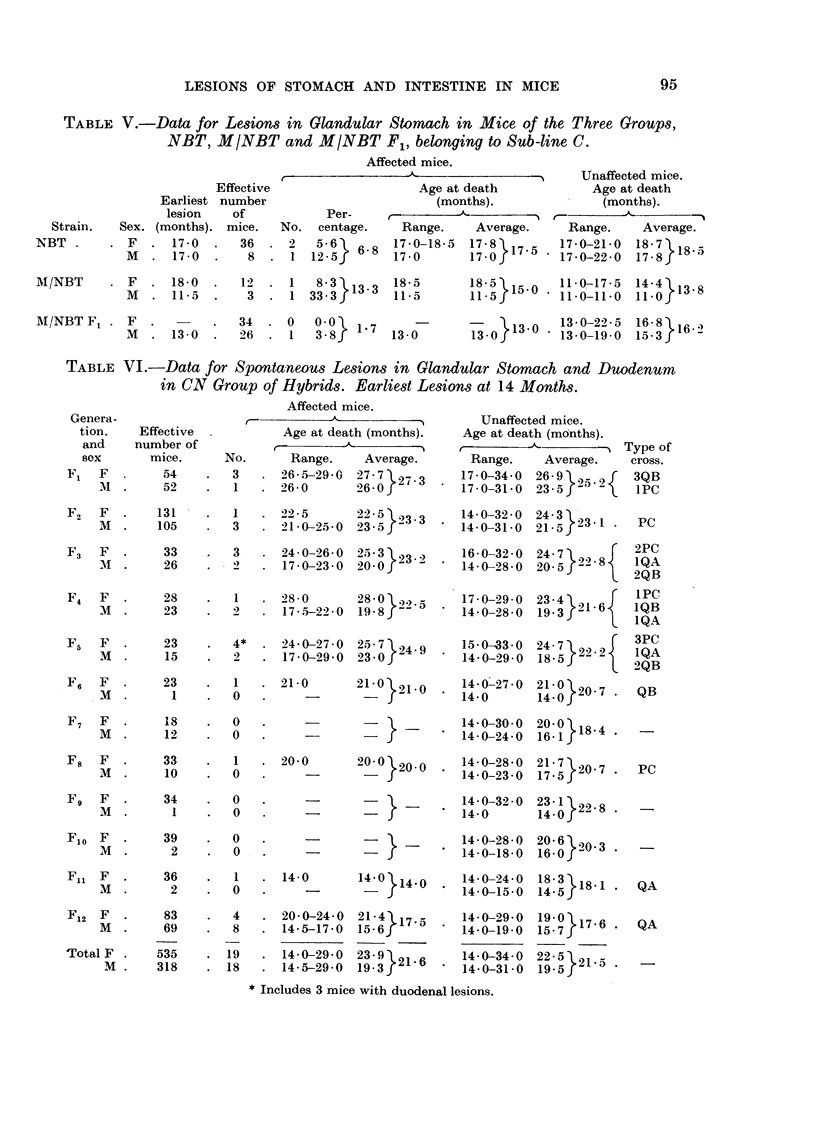

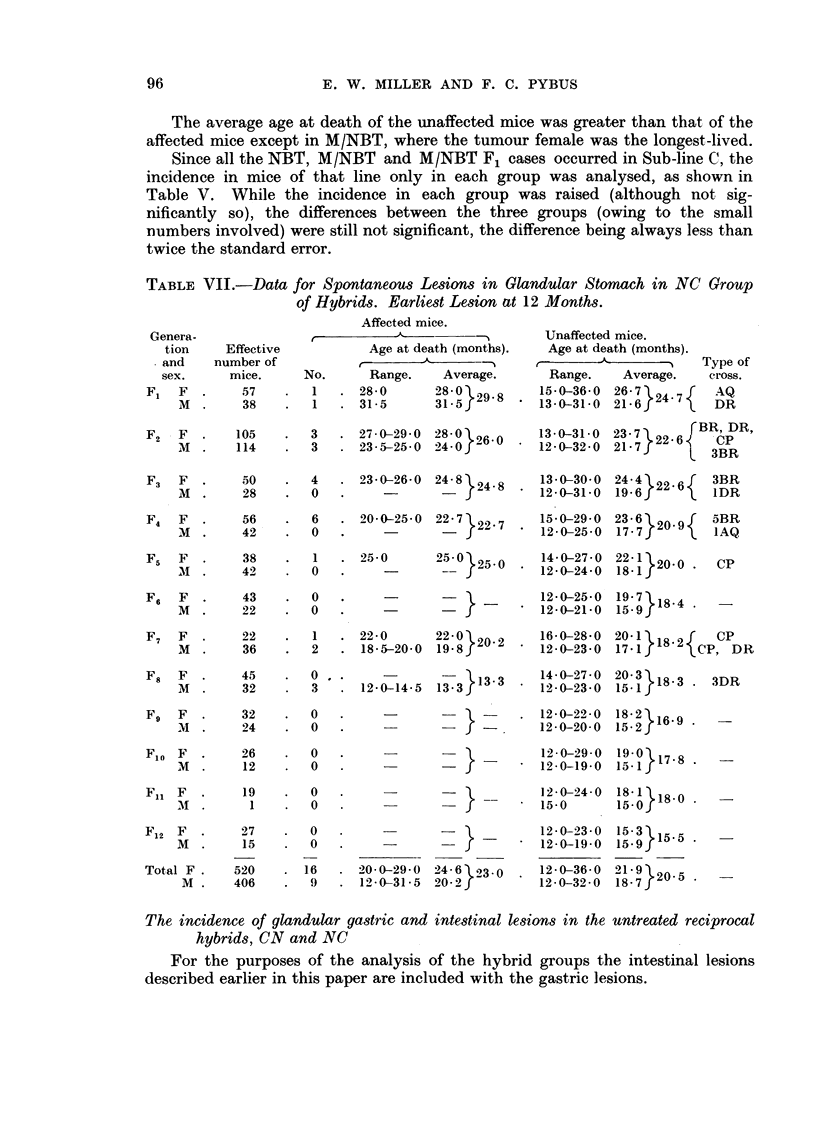

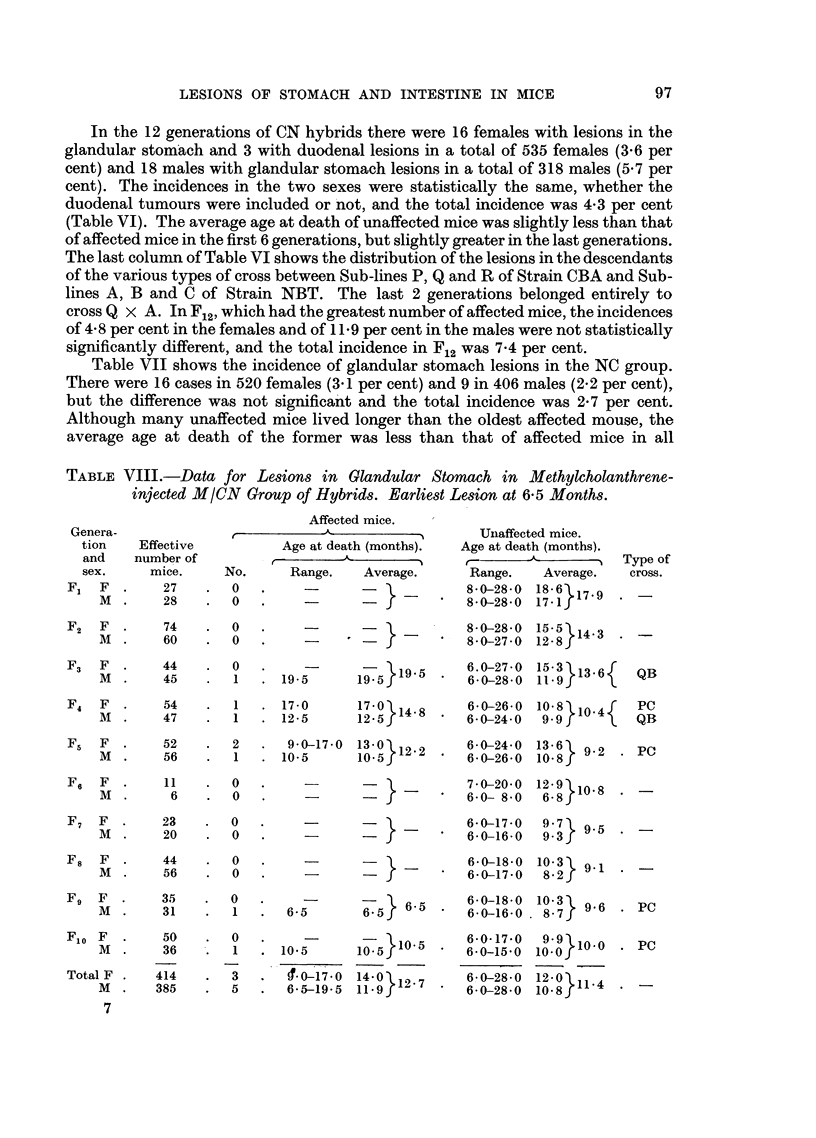

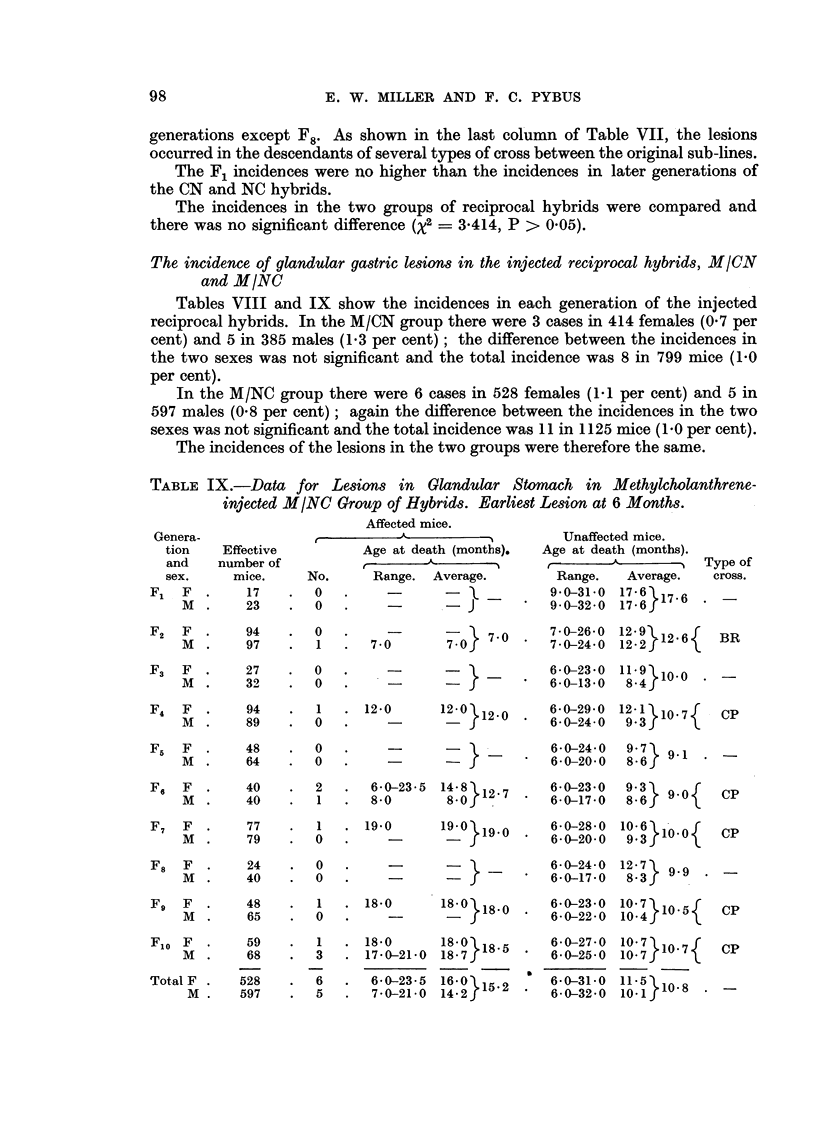

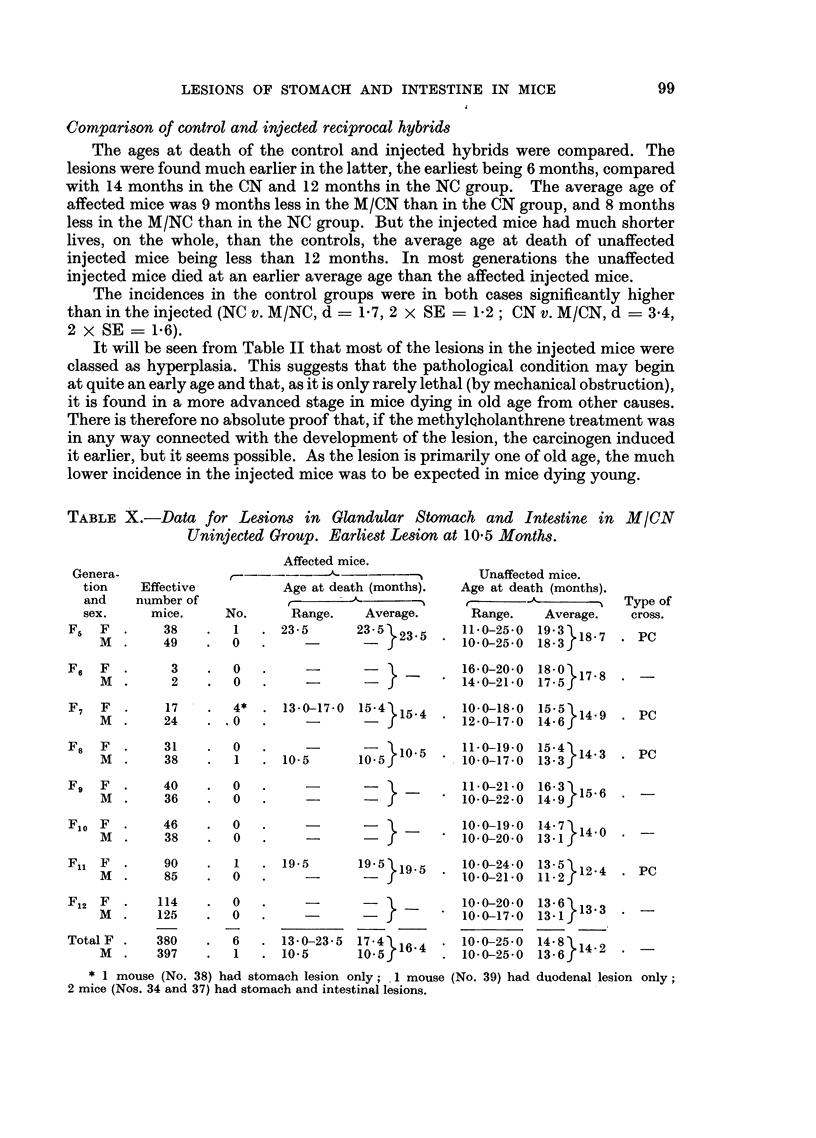

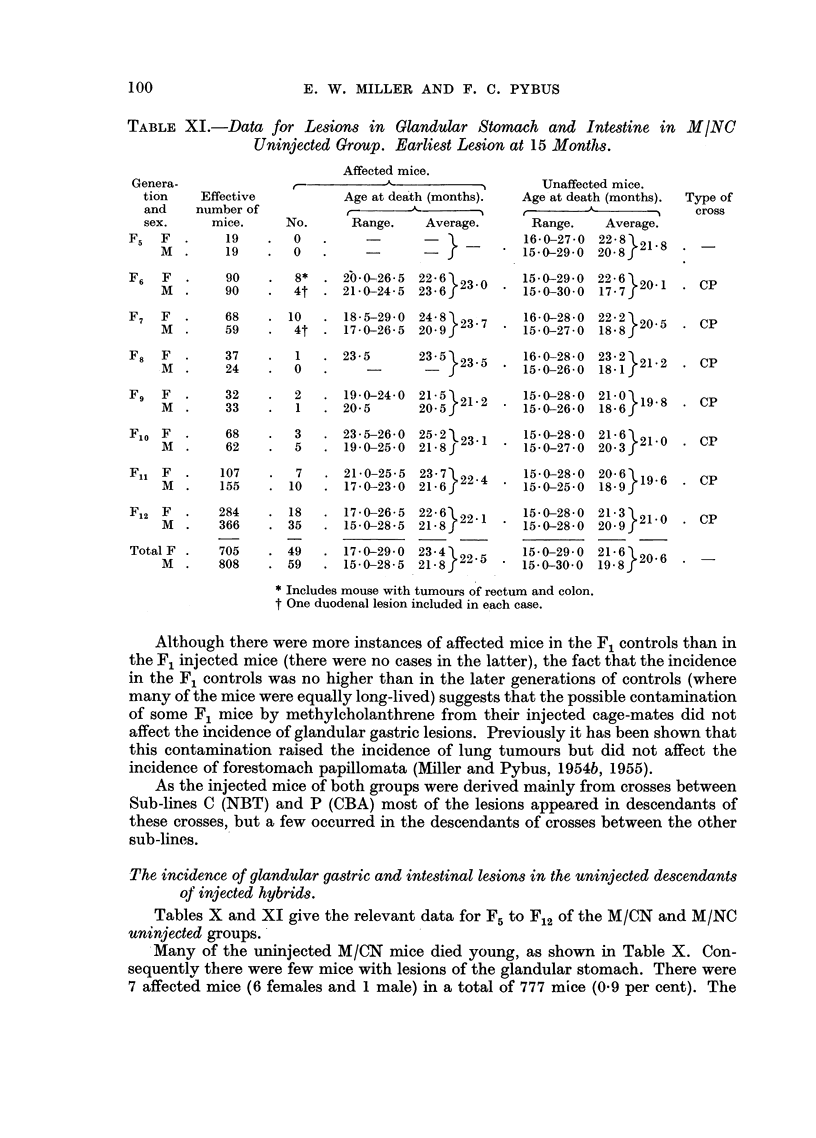

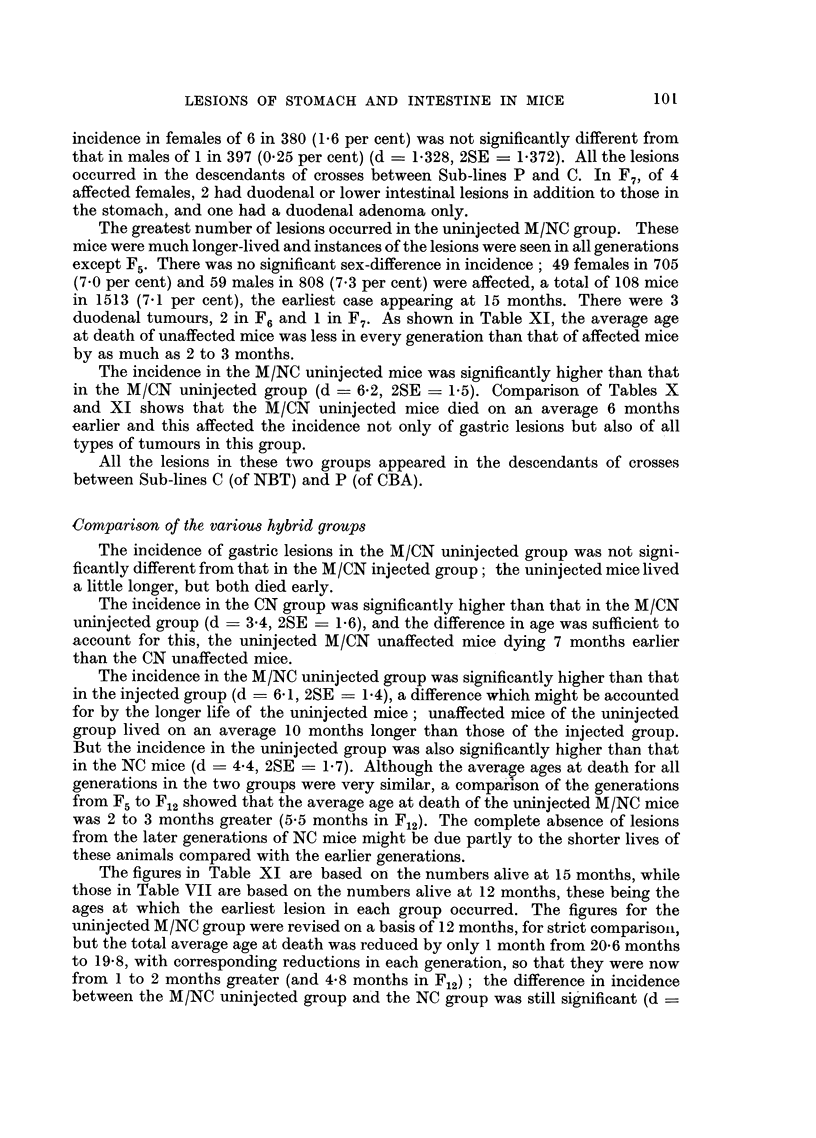

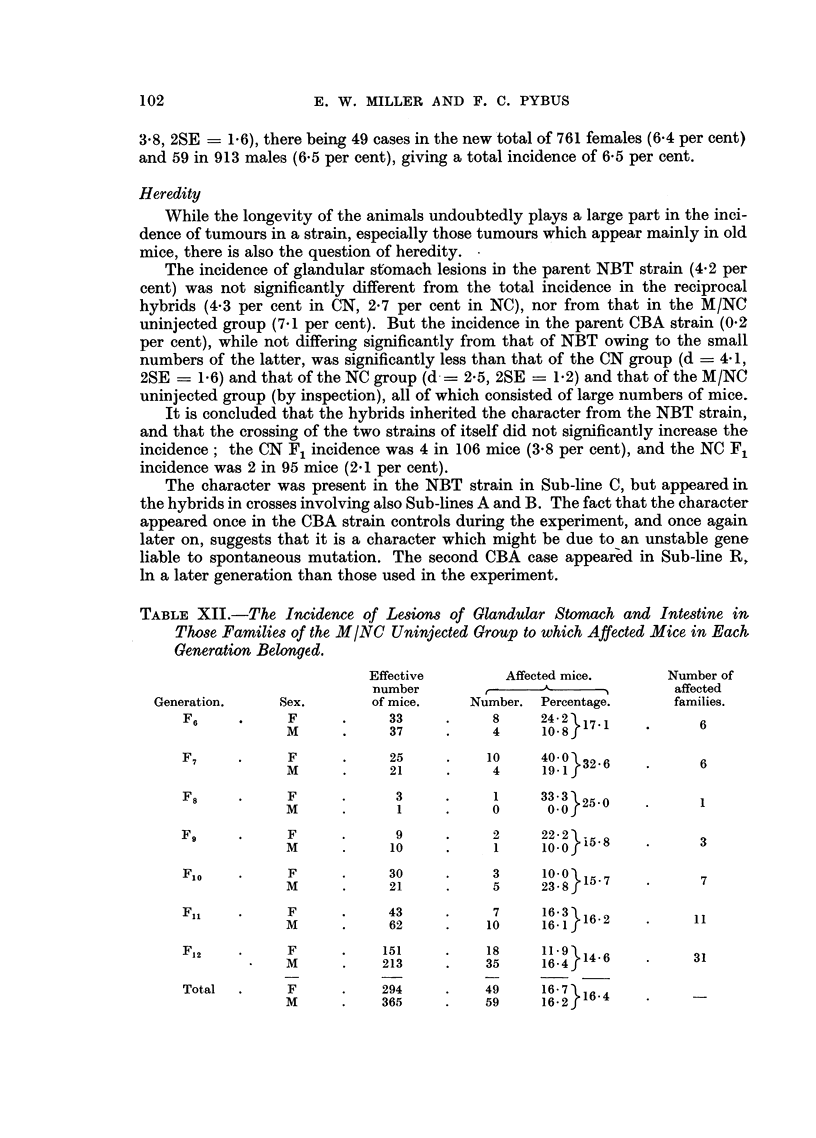

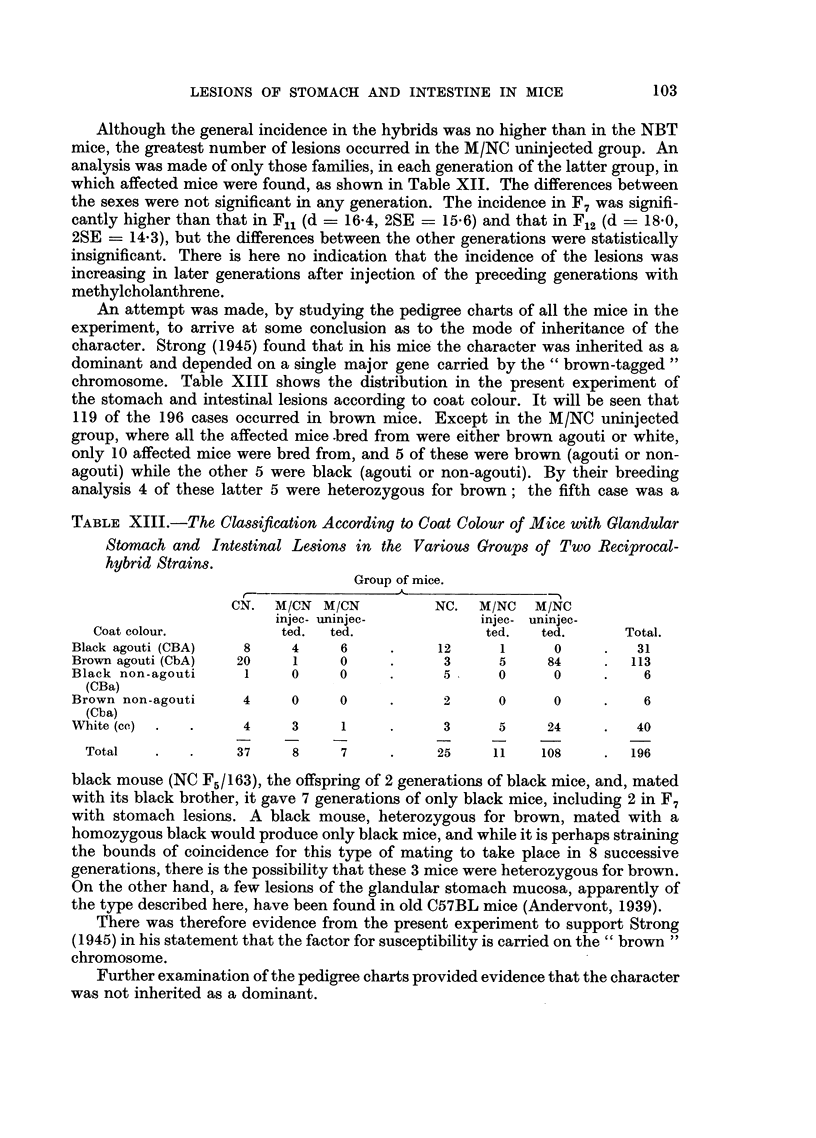

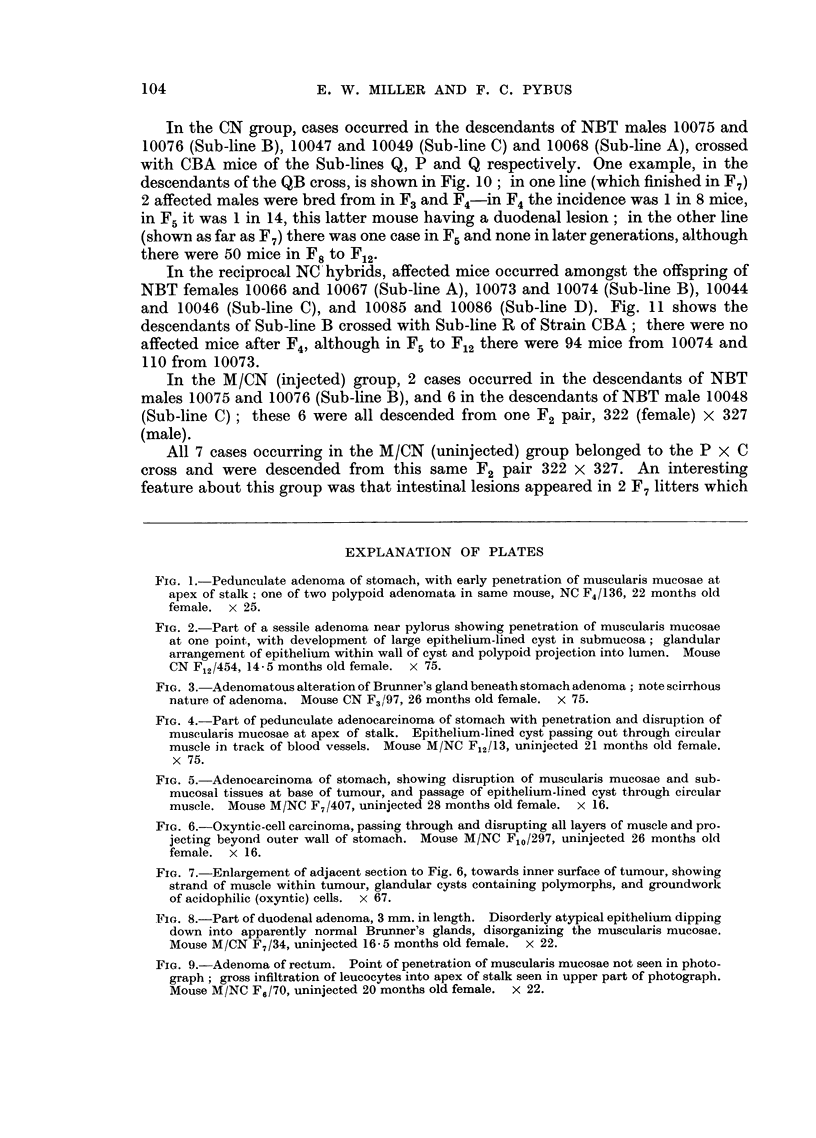

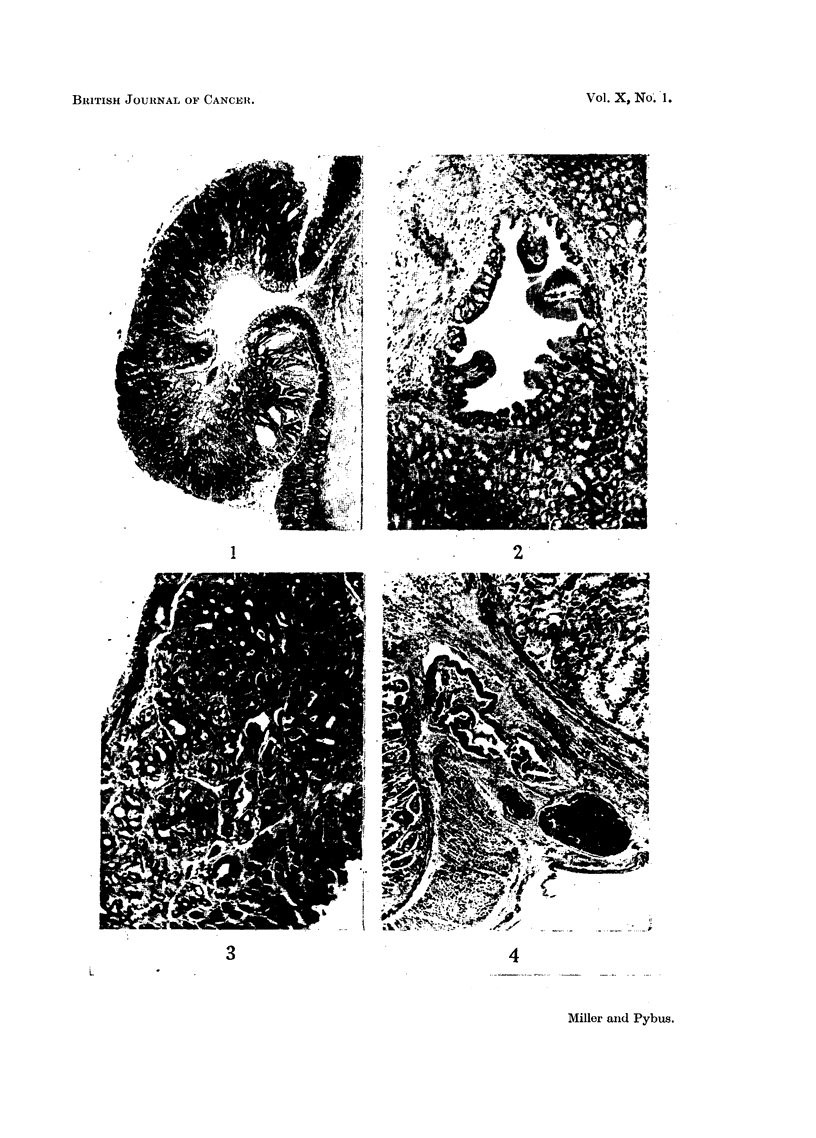

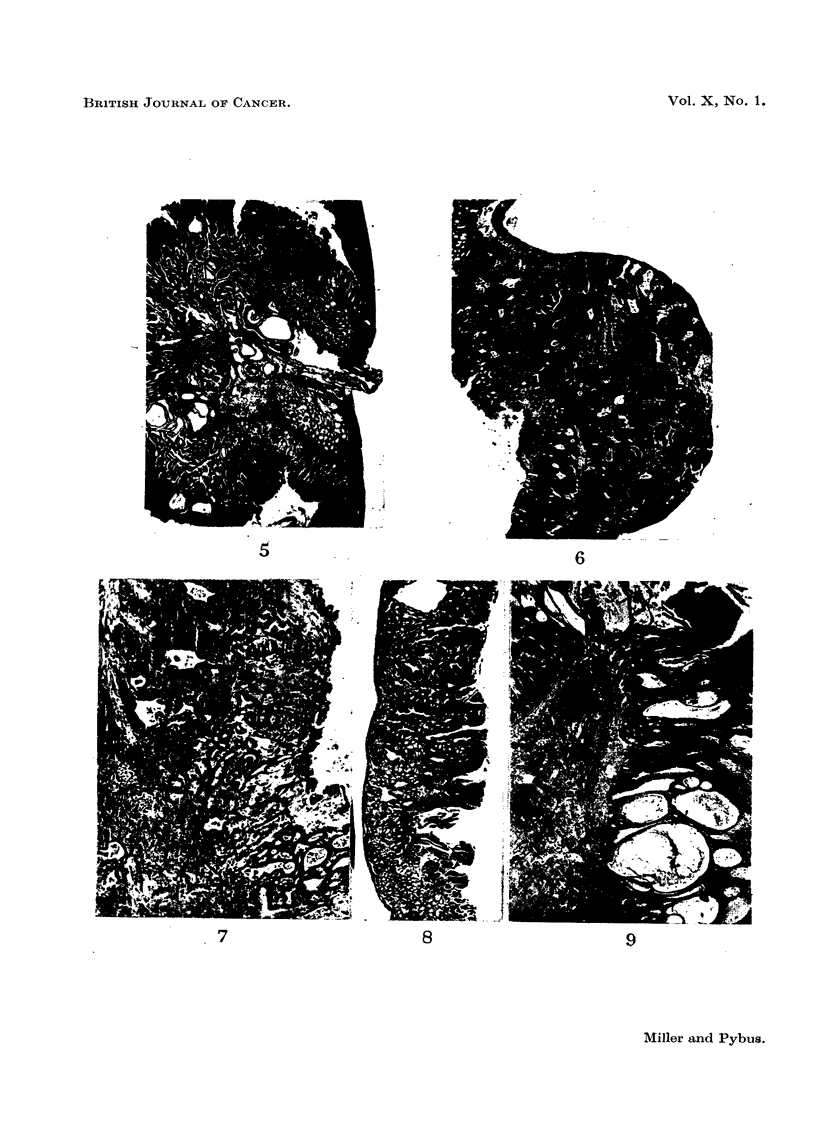

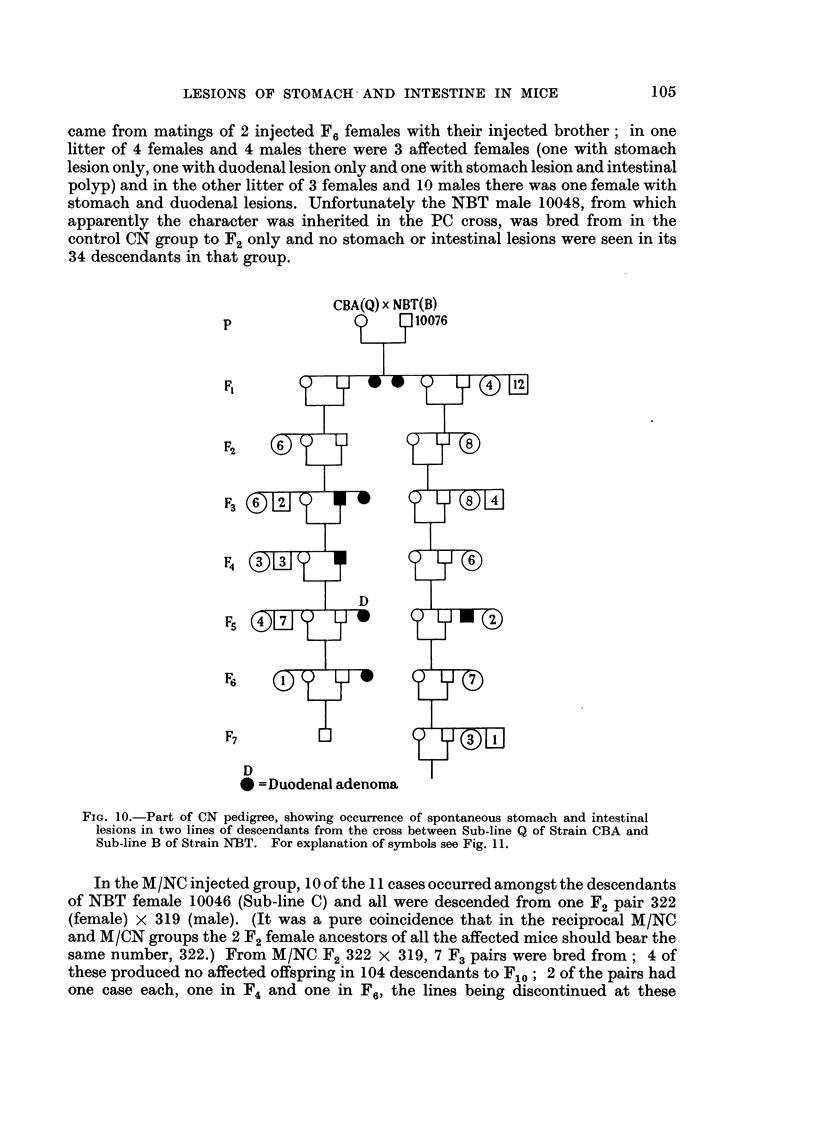

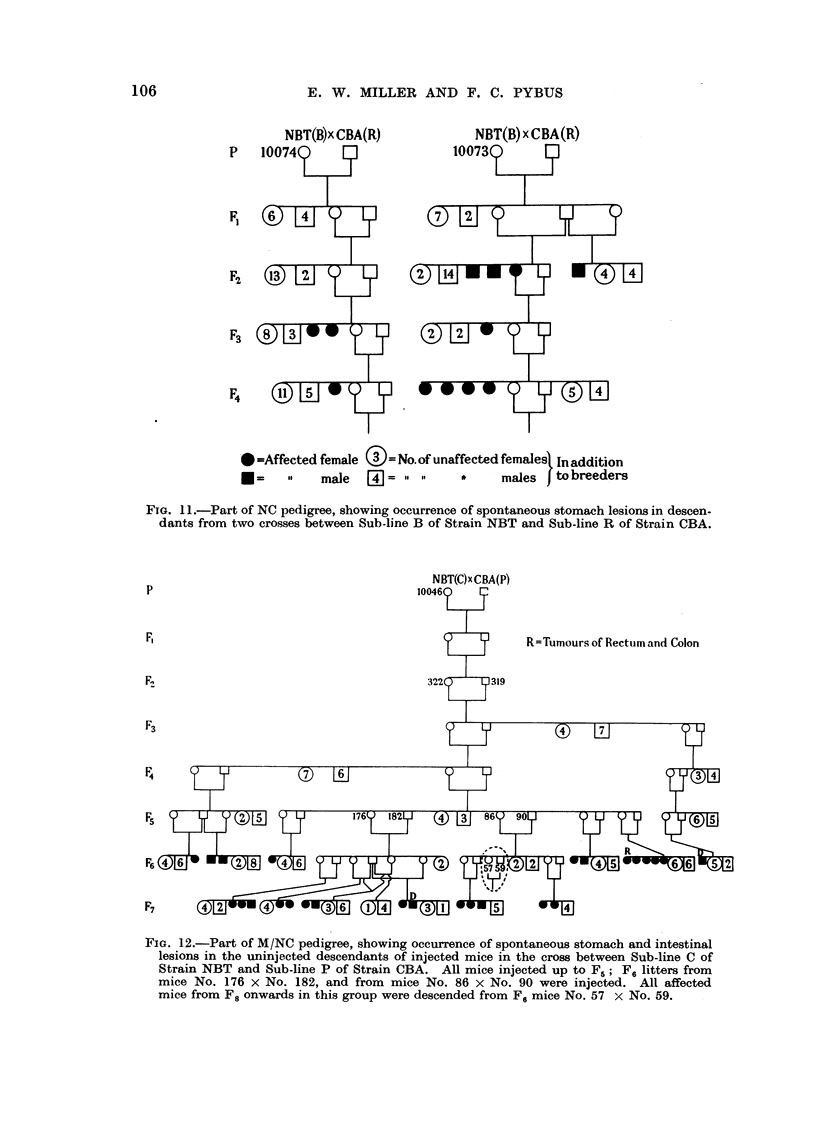

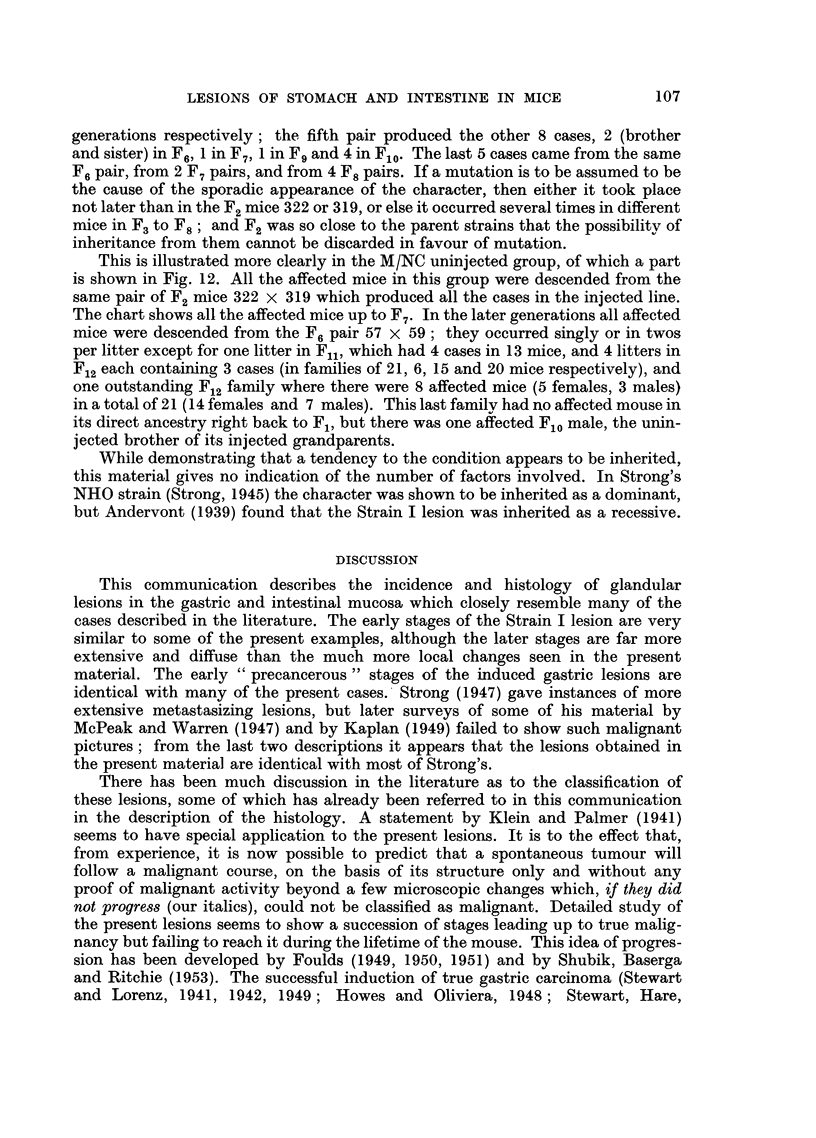

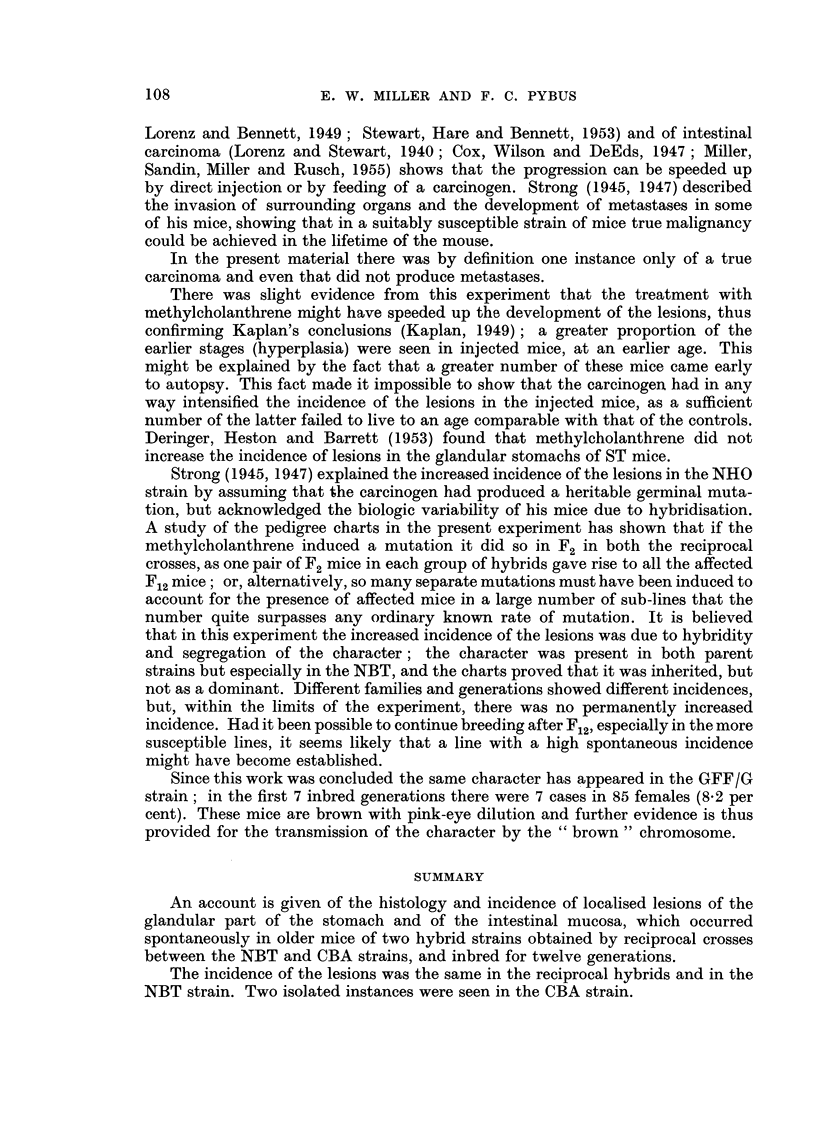

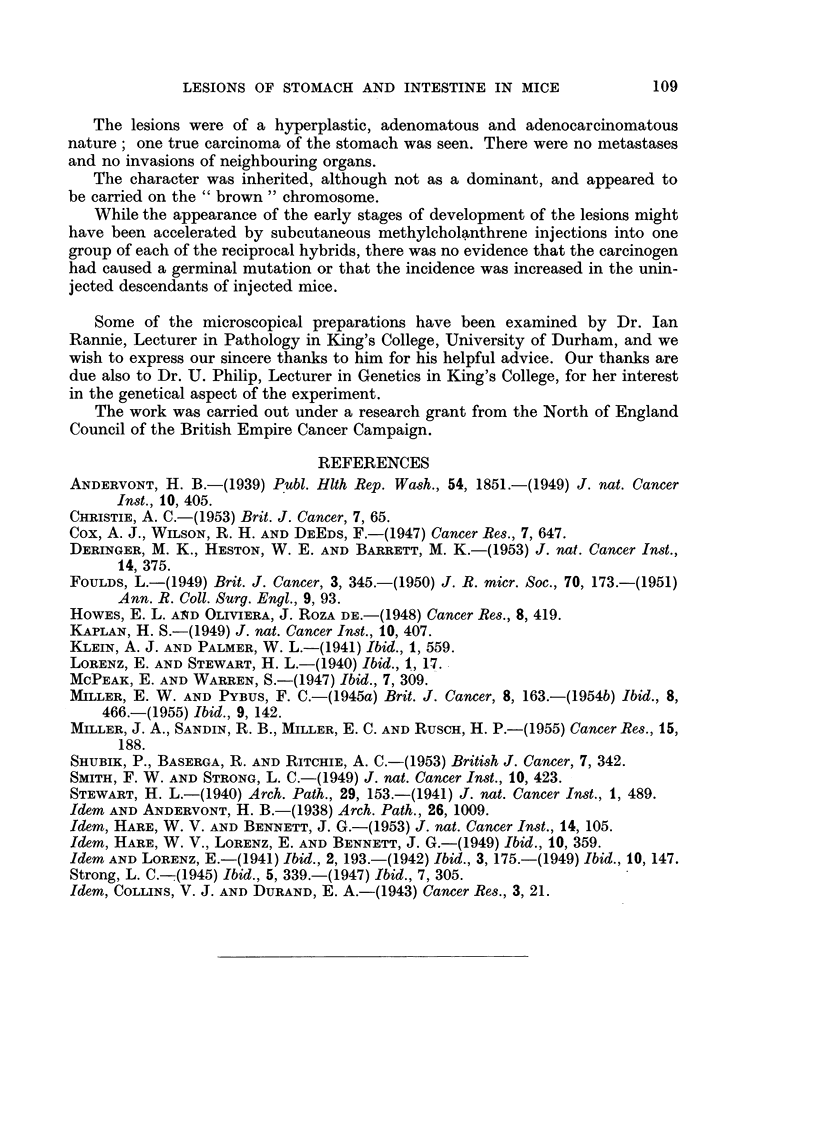


## References

[OCR_02076] ANDERVONT H. B. (1949). Spontaneous lesion of stomach in strain I mice.. J Natl Cancer Inst.

[OCR_02086] DERINGER M. K., HESTON W. E., BARRETT M. K. (1953). Spontaneous and induced tumors in strain ST mice.. J Natl Cancer Inst.

[OCR_02095] KAPLAN H. S. (1949). Lesions of the gastric mucosa in Strong strain NHO mice.. J Natl Cancer Inst.

[OCR_02101] MILLER E. W., PYBUS F. C. (1955). The incidence of spontaneous and induced forestomach tumours in mice of two inbred strains and their reciprocal hybrids.. Br J Cancer.

[OCR_02107] SHUBIK P., BASERGA R., RITCHIE A. C. (1953). The life and progression of induced skin tumors in mice.. Br J Cancer.

[OCR_02108] SMITH F. W., STRONG L. C. (1949). Studies on gastric neoplasia in mice; the histogenesis and influence of some endocrine factors.. J Natl Cancer Inst.

[OCR_02113] STEWART H. L., HARE W. V., BENNETT J. G. (1953). Tumors of the glandular stomach induced in mice of six strains by intramural injection of 20-methylcholanthrene.. J Natl Cancer Inst.

